# BicNET: Flexible module discovery in large-scale biological networks using biclustering

**DOI:** 10.1186/s13015-016-0074-8

**Published:** 2016-05-20

**Authors:** Rui Henriques, Sara C. Madeira

**Affiliations:** INESC-ID and Instituto Superior Técnico, Universidade de Lisboa, Lisboa, Portugal

**Keywords:** Fleixble module discovery, Large-scale biological networks, Biclustering

## Abstract

**Background:**

Despite the recognized importance of module discovery in biological networks to enhance our understanding of complex biological systems, existing methods generally suffer from two major drawbacks. First, there is a focus on modules where biological entities are strongly connected, leading to the discovery of trivial/well-known modules and to the inaccurate exclusion of biological entities with subtler yet relevant roles. Second, there is a generalized intolerance towards different forms of noise, including uncertainty associated with less-studied biological entities (in the context of literature-driven networks) and experimental noise (in the context of data-driven networks). Although state-of-the-art biclustering algorithms are able to discover modules with varying coherency and robustness to noise, their application for the discovery of non-dense modules in biological networks has been poorly explored and it is further challenged by efficiency bottlenecks.

**Methods:**

This work proposes Biclustering NETworks (BicNET), a biclustering algorithm to discover non-trivial yet coherent modules in weighted biological networks with heightened efficiency. Three major contributions are provided. First, we motivate the relevance of discovering network modules given by constant, symmetric, plaid and order-preserving biclustering models. Second, we propose an algorithm to discover these modules and to robustly handle noisy and missing interactions. Finally, we provide new searches to tackle time and memory bottlenecks by effectively exploring the inherent structural sparsity of network data.

**Results:**

Results in synthetic network data confirm the soundness, efficiency and superiority of BicNET. The application of BicNET on protein interaction and gene interaction networks from yeast,* E. coli* and Human reveals new modules with heightened biological significance.

**Conclusions:**

BicNET is, to our knowledge, the first method enabling the efficient unsupervised analysis of large-scale network data for the discovery of coherent modules with parameterizable homogeneity.

## Introduction

The increasing availability of precise and complete biological networks from diverse organisms provides an unprecedented opportunity to understand the organization and dynamics of cell functions [1]. In particular, the discovery of modules in biological networks has been largely proposed to characterize, discriminate and predict such biological functions [1–6]. The task of discovering modules can be mapped as the discovery of coherent regions in weighted graphs, where nodes represent the molecular units (typically genes, proteins or metabolites) and the scored edges represent the strength of interactions between the biological entities. In this context, a large focus has been placed on the identification of dense regions [7–10], where each region is given by a statistically significant set of highly interconnected nodes. In recent years, several biclustering algorithms have been proposed to discover dense regions from (bipartite) graphs by mapping them as adjacency matrices and searching for dense submatrices [8, 10–13]. A bicluster is then given by two subsets of strongly connected nodes.

Despite the relevance of biclustering to model local interactions [14, 15], the focus on dense regions comes with key drawbacks. First, such regions are associated with either trivial or well-known (putative) modules. Second, the scores of the interactions associated with less studied genes, proteins and metabolites have lower confidence (being the severity of these penalizations highly dependent on the studied organism) and may not reflect the true role of these molecular interactions in certain cellular processes [16]. In particular, the presence of (well-studied) regular/background cellular processes may mask the discovery of sporadic or less-trivial processes, preventing the discovery of new putative functional modules.

Although biclustering has been proved to be an effective tool to retrieve exhaustive structures of dense regions in a network [8, 11–13, 17], it has not yet been effectively applied to the discovery of modules with alternative forms of coherency due to two major challenges. First, despite the hypothesized importance of discovering biclusters associated with non-dense regions (characterized for instance by constant, order-preserving or plaid coherencies), there are not yet mappings enabling the understanding of their biological meaning. Second, the hard combinatorial nature of biclustering data when considering non-dense forms of coherency, together with the high dimensionality of the adjacency matrices derived from biological networks, are often associated with memory and time bottlenecks, and/or undesirable restrictions on the structure and quality of biclusters.

This work aims to tackle these problems by: (1) analyzing the biological relevance of modeling non-dense regions in a biological network, and (2) enabling the efficient discovery of flexible biclustering solutions from large-scale networks. For this end, we propose the algorithm Biclustering NETworks (BicNET). BicNET integrates principles from pattern-based biclustering algorithms [15, 18] and adapts their data structures and searches to explore efficiency gains from the inherent sparsity of biological networks. Furthermore, we motivate the relevance of finding non-dense yet coherent modules and provide a meaningful analysis of BicNET’s outputs. In this context, this paper has six major contributions:

- Principles for the discovery of modules in weighted graphs given by parameterizable forms of coherency (including constant, order-preserving, symmetric assumptions) with non-dense yet meaningful interactions, and given by plaid structures to accommodate weight variations explained by the network topology;
- Principles for the discovery of modules robust to missing and noisy interactions;
- New biclustering algorithm (BicNET) able to accommodate the proposed principles and adequately discover modules from data with arbitrary-high sparsity;
- Adequate data structures and searches to guarantee BicNET’s applicability over large networks;
- Principles for biclustering different types of networks, including homogeneous and heterogeneous networks, and networks with either weighted or labeled interactions;
- Theoretical and empirical evidence of the biological relevance of the modules discovered using non-dense coherency assumptions.

Results gathered from synthetic and real data demonstrate the relevance of the proposed principles for biclustering large-scale biological networks, and in particular the ability of BicNET to discover a complete set of non-trivial yet coherent and (biologically) significant modules from molecular-interactions inferred from knowledge repositories [16] and experimental data [19] for different organisms.

**Fig. 1:**
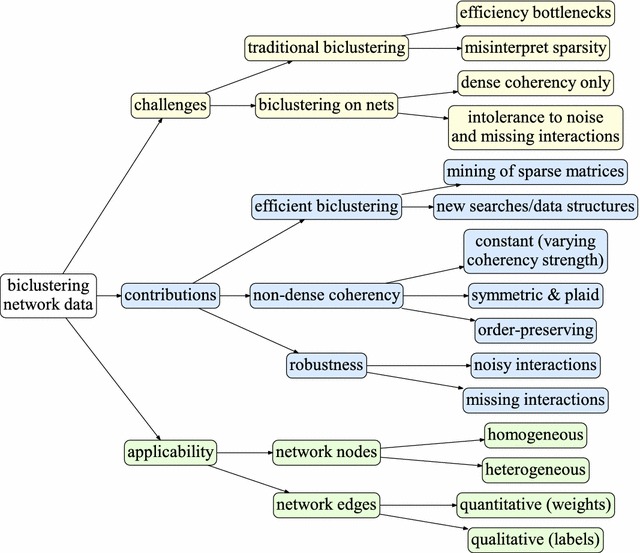
Structured view on the existing challenges, proposed contributions (and their applicability) for an effective and efficient (pattern-based) biclustering of network data

Figure 1 provides a structured view on the challenges and proposed contributions. Accordingly, this work is organized as follows. First, we provide background on the target task. "BicNET: solution" and "BicNET: algorithmic aspects" sections describe the principles used by BicNET and its algorithmic details. "Results and discussion" section provides empirical evidence for the relevance of BicNET to unravel non-trivial yet relevant modules in synthetic and real biological networks. Finally, we draw conclusions and highlight directions for future work.

## Background

In this section, we provide the basics on biological networks, background on biclustering network data, and a discussion on the importance and open challenges of biclustering non-dense network modules. Finally, the opportunities and limitations of pattern-based biclustering for this end are surveyed.

### Biological networks

A biological network is a linked collection of biological entities (proteins, protein complexes, genes, metabolites, etc.). Biological networks are typically classified according to the observed type of biological entities and their homogeneity. Homogeneous networks are given, for instance, by protein-protein interactions (PPI) and gene interactions (GI). Heteregeneous networks capture interactions between two distinct data sources, such as proteins and protein complexes, host and viral molecules, biological entities and certain functions, among others. Biological networks can be further classified according to the type of interactions: weighted interactions (either determining the degree of physical or functional association) or qualitative/labeled interactions (such as ’binding’, ’activation’ and ’repression’, etc.). The methods targeted by this work aim to analyze both homogeneous and heterogeneous biological networks with either weighted or qualitative interactions.

### Biclustering network data

The introduced types of biological networks can be mapped as bipartite graphs for the subsequent discovery of modules.

#### **Definition 1**

A graph is defined by a set of nodes *X *= $\{x_1,..,x_n\}$, and interactions $a_{ij}$ relating nodes $x_i$ and $x_j$, either numeric ($a_{ij}\in \mathbb {R}$) or categoric ($a_{ij}\in \mathcal {L}$, where $\mathcal {L}$ is a set of symbols). A bipartite graph is defined by two sets of nodes *X* = $\{x_1,\ldots,x_n\}$ and *Y* = $\{y_1,\ldots,y_m\}$ with interactions $a_{ij}$ between nodes $x_i$ and $y_j$.

#### **Definition 2**

Given a bipartite graph (*X*, *Y*), the **biclustering task** aims to identify a set of biclusters $\mathcal {B}$ = $\{B_1,..,B_p\}$, where each bicluster $B_k$ = $(I_k,J_k)$ is a module (or subgraph) in the graph given by two subsets of nodes, $I_k\subseteq X\wedge J_k\subseteq X$, satisfying specific criteria of *homogeneity* and statistical significance.

**Fig. 2:**
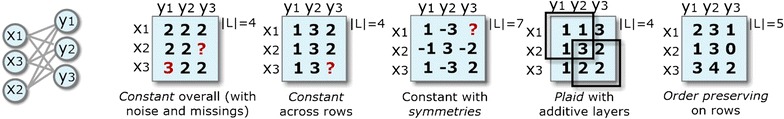
Illustrative discrete biclusters with varying coherency and quality

**Fig. 3:**
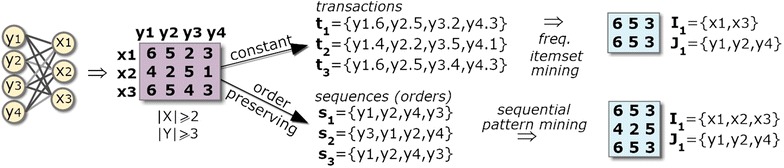
Pattern-based discovery of biclusters with constant and order-preserving coherency

Under the previous definitions, both homogeneous networks (*Y* = *X*) and heterogeneous networks are candidates for biclustering. The task of biclustering network data can be tackled by using the traditional task of biclustering real-valued matrices by subsequently mapping a bipartite graph as a matrix (with rows and columns given by the nodes and values given by the scored interactions). In this case, subsets of rows and columns define a bicluster. A bicluster is associated with a module in the network with coherent interactions (see Figs. 2, 3).

The **homogeneity** criteria determines the structure, coherency and quality of the biclustering solutions, while the *statistical significance* of a bicluster determines whether its probability of occurrence deviates from expectations. The homogeneity of a biclustering model is commonly guaranteed through a merit function. An illustrative merit function is the variance of the values in the bicluster. The *structure* of a biclustering solution is essentially defined by the number, size and positioning of biclusters. Flexible structures are characterized by an arbitrary-high set of (possibly overlapping) biclusters. The *coherency* of a bicluster is defined by the observed correlation of values (coherency assumption) and by the allowed deviation from expectations (coherency strength). The *quality* of a bicluster is defined by the type and amount of accommodated noise. Figure 2 illustrates biclusters with varying coherency and quality.

The paradigmatic assumption when biclustering network data is to rely on the dense coherency [20] (Definition 3). Definitions 4 and 5 formalize for the first time the meaning of distinct coherency assumptions in the context of weighted network data. The constant assumption (Definition 4) introduces the possibility of accommodating biological entities with (possibly) distinct strengths/types of interactions yet coherent behavior. This already represents an improvement in terms of flexibility against the dense assumption. Alternative coherency assumptions can be given by symmetric, order-preserving and plaid models (Definition 5).

#### **Definition 3**

Let the elements in a bicluster $a_{ij}\in (I,J)$ have a specific coherency. A bicluster is **dense** when the average of its values is significantly high (deviates from expectations), where the average value is given by $\frac{1}{|I||J|}\Sigma _{i\in I}\Sigma _{j\in J} a_{ij}.$

#### **Definition 4**

A **constant** coherency assumption is observed when $a_{ij}=k_j+\eta _{ij}$, where $k_j$ is the expected strength of interactions between nodes in *X* and $y_j$ node from *Y* and $\eta _{ij}$ is the noise factor. In other words, constant biclusters have similarly scored interactions for each node from one of the two subsets of nodes. The *coherency strength* of a constant module is defined by the $\delta$ range, where $\eta _{ij}\in [-\delta /2,\delta /2]$.

#### **Definition 5**

The symmetric assumption considers the (possible) presence of **symmetries** within a constant bicluster, $a_{ij}=k_jc_i$+$\eta _{ij}$ where $c_i\in \{-1,1\}$. An **Order**-preserving assumption is verified when the values for each node in one subset of nodes of a bicluster induce the same linear ordering across the other subset of nodes. A **plaid** assumption [21] considers cumulative contributions on the elements where biclusters/subgraphs overlap.

### Pattern-based biclustering

The discovery of dense modules in biological networks has been mainly accomplished using pattern-based biclustering algorithms [8, 10–13, 17] due to their intrinsic ability to exhaustively discover flexible structures of biclusters. Despite the focus on dense biclusters, pattern-based biclustering is natively prepared to model alternative forms of coherency associated with constant models (when using frequent itemset mining) [15] and order-preserving models (when using sequential pattern mining) [22]. In this context, patterns (itemsets, rules, sequences or graphs appearing in a symbolic datasets with certain frequency) can be mapped as biclusters under a specific coherency strength determined by the number of symbols in the dataset ($\delta=1/|\mathcal {L}|$  where $\mathcal {L}$ is the alphabet of symbols). This mapping^1^ led to the development of several pattern-based approaches for biclustering [15, 22–24]. Figure 3 illustrates how pattern mining can be used to derive constant and order-preserving biclusters. Recent advances on pattern-based biclustering also show the possibility to discover biclusters according to symmetric and plaid models [15, 21] and to further guarantee their robustness to noise [15, 18, 22].

### Related work

A large number of algorithms has been proposed to find modules in unweighted graphs (binary interactions) and weighted graphs (real-valued interactions) mapped from biological networks. In the context of *unweighted graphs*, clique detection with Monte Carlo optimization [25], probabilistic motif discovery [26] and clustering on graphs [27] have been, respectively, applied to discover modules in PPIs (yeast), GIs (*E. coli*) and metabolic networks.

In unweighted bipartite graphs, the densest regions correspond to bicliques. Bicliques have been efficiently discovered using Motzkin-Straus optimization [9], density-constrained biclustering [28], formal concepts and pattern-based biclustering [11, 12, 17]. In the context of *weighted graphs*, the density of a module is given by the average weight of the interactions within the module. Different scores have been proposed to determine the weight of an interaction, including the: functional correlation between biological entities (when interactions are predicted from literature or other knowledge-based sources); or physical association (when interactions are derived from experimental data based for instance on the correlated variation of the expression of genes or concentration of molecular compounds). Modules given by densely connected subgraphs have been discovered from PPIs using betweenness-based partitioning [27] and flow-based clustering algorithms in graphs [29]. Biclustering has been largely applied for this end^2^ using SAMBA [20], multi-objective searches [34] and pattern-based biclustering [6, 8, 10]. The application of these methods over both homogeneous and viral-host PPIs show that protein complexes largely match the found modules [27, 29, 34].

Pattern-based biclustering has been largely applied for the discovery of dense network modules [6, 8, 10–13, 17] due to their intrinsic ability to exhaustively discover flexible structures of biclusters. In unweighted graphs, closed frequent itemset mining and association rule mining were applied to study interactions between proteins and protein complexes in yeast proteome network [12, 17] and between HIV-1 and human proteins to predict and characterize host-cellular functions and their perturbations [12, 13]. More recently, association rules were also used to obtain a modular decomposition of GI networks with positive and negative interactions ($a_{ij}\in${−1,0,1}) [11] for understanding between-pathway and within-pathway models of GIs. In weighted graphs, Dao et. al [6] and Atluri et. al [10] relied on the loose antimonotone property of density to propose weight-sensitive pattern mining searches. DECOB [8], originally applied to PPIs and GIs from human and yeast, uses an additional filtering step to output dissimilar modules only.

Some of the surveyed contributions have been used or extended for classification tasks such as function prediction [2, 12, 13]. Discriminative modules, often referred as multigenic markers, are critical to surpass the limitations of single gene markers and topological markers [2, 6, 35, 36]. Network-based (bi)clustering methods for function prediction have been comprehensively reviewed by Sharan et al. [2].

The problem with the surveyed contributions is their inability to discover modules with parameterizable coherency assumption and strength.

Some simple variants of the dense coherency assumption have been reviewed by Dittrich et al. [37], Ideker et al. [4] and Sharan et al. [2]. Yet, the studied algorithms do not support the coherency assumptions explored in this work (Definitions 4 and 5). A first attempt to apply biclustering algorithms with non-dense coherency over biological networks was presented by Tomaino et al. [40]. Despite its disruptive nature, this work suffers from two drawbacks. First, only considers very small PPIs (human and yeast PPIs with less than 200 interactions) due to the scalability limits of the surveyed biclustering algorithms to handle high-dimensional adjacency matrices. Second, although enriched biological terms have been identified for the discovered modules (pointing out the importance of using non-dense forms of coherency), an in-depth analysis of the modules with enriched terms as well as an explanation of the meaning of their coherency in the assessed networks is absent.

### Research questions

Although biclustering can be easily applied over biological networks to discover biclusters with varying coherency criteria, three major challenges have been preventing this possibility up to date. First, state-of-the-art biclustering algorithms are not able to scale for the majority of the available biological networks due to the high dimensionality of the mapped matrices [41]. Second, non-dense forms of coherency often come with the cost of undesirable restrictions on the number, positioning (e.g. non-overlapping condition) and quality of biclusters [15]. Finally, there is a generalized lack of understanding of the relevance and biological meaning associated with non-dense modules [41]. Although pattern-based biclustering can be used to address the second challenge [15], it still presents efficiency bottlenecks and further knowledge is required for the correct interpretation of these regions.

In this context, this work targets two major research problems:

- Discussion on whether biclustering can be efficiently and consistently applied over large-scale biological networks for the discovery of non-dense modules;
- Assessment of the biological relevance of discovering network modules with varying coherency criteria.

## BicNET: solution

In this section, we first introduce principles to enable the sound application of (pattern-based) biclustering over network data. Second, we motivate the relevance of discovering coherent modules following constant, symmetric and plaid models. Third, we show how to discover modules robust to noisy and missing interactions. Fourth, we extend pattern-based searches to seize efficiency gains from the inherent structural sparsity of biological networks. Fifth, we see how module discovery can be guided in the presence of domain knowledge. Finally, we overview the opportunities of pattern-based biclustering biological networks.

### Biclustering network data

**Fig. 4:**
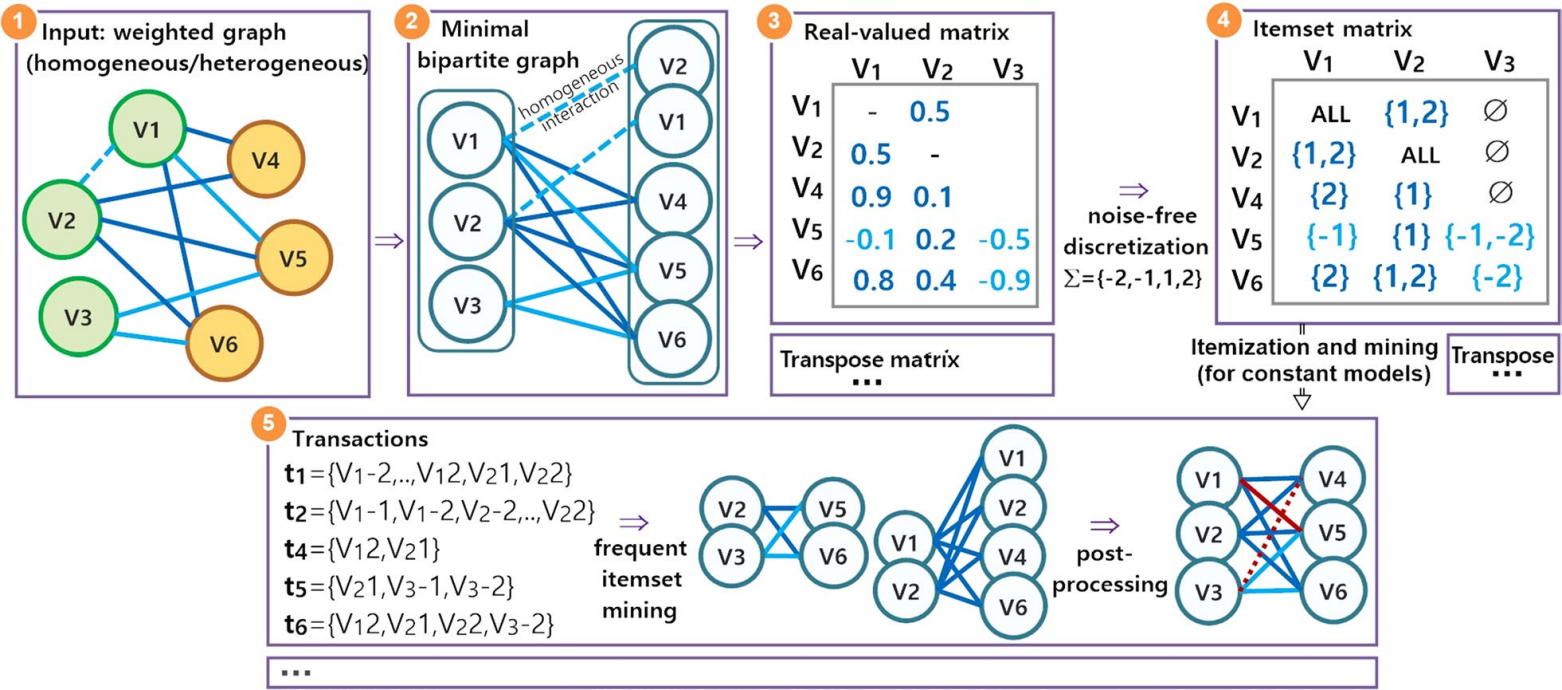
Pattern-based biclustering of (heterogeneous) biological networks using real-valued matrices derived from minimal weighted bipartite graphs

For an effective application of state-of-the-art biclustering algorithms towards (weighted) graphs derived from network data, two principles should be satisfied. First, the weighted graph should be mapped into a minimal bipartite graph. In heterogeneous networks, multiple bipartite graphs can be created (each with two disjoint sets of nodes with heterogeneous interactions). The minimality requirement can be satisfied by identifying subsets of nodes with cross-set interactions but without intra-set interactions to avoid unnecessary duplicated nodes in the disjoint sets of nodes (see Fig. 4). This is essential to avoid the generation of large bipartite graphs and subsequent very large matrices. Second, when targeting non-dense coherencies from homogeneous networks, a real-valued adjacency matrix is derived from the bipartite graph by filling both $a_{ij}$ and $a_{ji}$ elements with the value of the interaction between $x_i$ and $x_j$ nodes. In the context of an heterogeneous network, two real-valued adjacency matrices are derived: one matrix with rows and columns mapped from the disjoint sets of nodes and its transpose. Despite the relevance of this second principle, some of the few attempts to find non-dense biclusters in biological networks fail to satisfy it [40], thus delivering incomplete and often inconsistent solutions.

Under the satisfaction of the previous two principles, a wide-range of biclustering algorithms can be applied to discover modules with varying forms of coherency [14]. Yet, only pattern-based biclustering [15, 18, 42] is able to guarantee the discovery of flexible structures of biclusters with parameterizable coherency and quality criteria. Additionally, pattern-based biclustering provides an environment to easily measure the relevance and impact of discovering modules with varying coherency and tolerance to noise.

In particular, we rely on BicPAM, BiP and BicSPAM algorithms [15, 21, 22], which respectively use frequent itemset mining, association rule mining and sequential pattern mining to find biclusters with constant, plaid and order-preserving coherencies (in both the absence and presence of symmetries). These algorithms integrate the dispersed contributions from previous pattern-based algorithms and address some of their limitations, providing key principles to: (1) surpass discretization problems by introducing the possibility to assign multiple discrete values to a single element; (2) accommodate meaningful constraints and relaxations, while seizing their efficiency gains; and (3) robustly handle noise and missing values.

Figure 4 provides a view on how transactions can be derived from (heterogeneous) network data for the discovery of constant modules based on the itemization (preceded by a noise-free discretization) of the (bipartite) graph. A detailed description and formalization of these procedures and subsequent pattern mining and postprocessing steps is provided in [15, 22].

### Modules with non-dense forms of coherency using pattern-based biclustering

#### Constant model

**Fig. 5:**
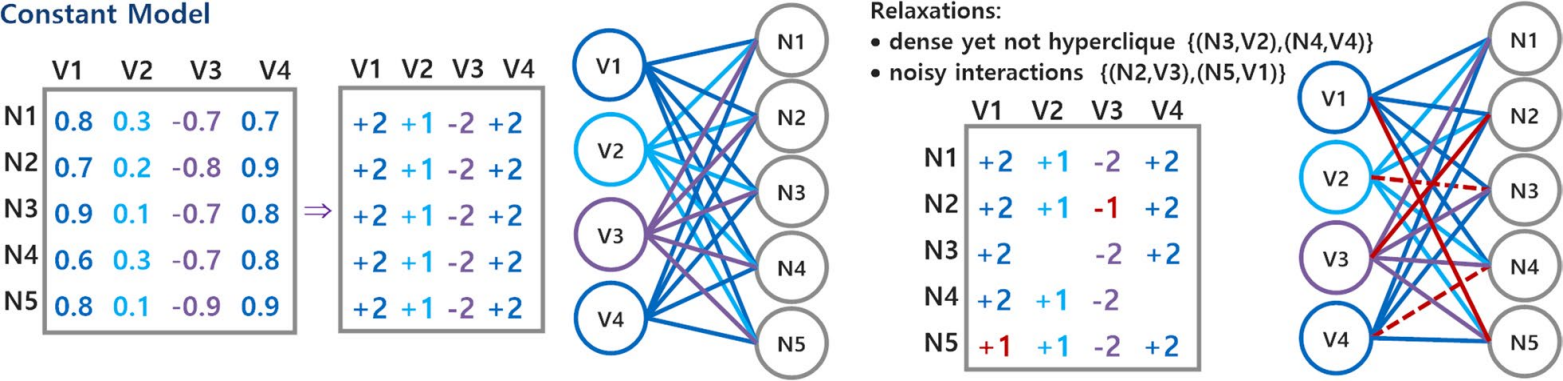
Biclustering non-dense modules: the constant model and the relevance of tolerating noise

Given a bicluster defining a module with coherent interactions between two sets of nodes, the constant coherency (Definition 4) requires the nodes in one set to show a single type of interaction with the nodes in the other set. The constant model is essential to model biological entities with possibly distinct (yet coherent) responsiveness, influence or role in a given module. Despite the inherent simplicity of the constant model, its application over biological networks has not been previously targeted. To illustrate the relevance of the constant model, consider a biological network with a set of interactions between genes and proteins, where their absolute weight defines the strength of the association and their sign determines whether the association corresponds to activation or repression mechanisms. The constant model guarantees that when a gene is associated with a group of proteins, it establishes the same type of interaction with all these proteins (such as heightened activation of the transcription of a complex of proteins). When analyzing the transposed matrix (by switching the disjoint sets of the bipartite graph), similar relations can be observed: a protein coherently affects a set of genes (softly repressing their expression, for example). The constant model can also disclose relevant interactions between homogeneous groups of genes, proteins and metabolites. Figure 5 provides an illustrative constant module.

The proposed constant model can be directly applied to networks with qualitative interactions capturing distinct types of regulatory relations, such as *binding*, *activation* or *enhancement* associations. Qualitative interactions are commonly observed for a wide-variety of PPIs [12, 13].

The constant model is essential to guarantee that biological entities with non-necessarily high (yet coherent) influence on another set of entities are not excluded. Typically, the constant coherency leads to the discovery of larger modules than the dense coherency. The exception is when the dense coherency is not given by highly weighted interactions, but instead by all interactions independently of their weight (extent of interconnected nodes). In this context, dense modules can be larger than constant modules.

#### Symmetric model

**Fig. 6:**
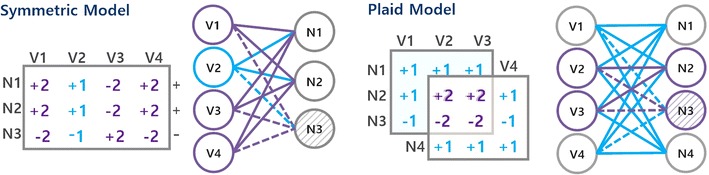
Non-dense biclustering modules: the symmetric and plaid models

The presence of symmetries is key to simultaneously capture activation and repression mechanisms associated with the interactions of a single node [15]. The symmetric model introduces a new degree of flexibility by enabling the discovery of more complex regulatory modules, where a specific gene/protein may positively regulate some genes/proteins and negatively regulate other genes/proteins within a single module, yet still respect the observed coherency. Figure 6 (left) illustrates the symmetric model, where symmetries (identified with dashed lines) are verified on rows.

#### Plaid model

The plaid assumption [21] is essential to describe overlapping regulatory influence associated with cumulative effects in the interactions between the nodes in a biological network. Illustrating, consider that two genes interact in the context of multiple biological processes, a plaid model can consider their cumulative effect on the score of their interaction based on the expected score associated with each active process. The same observation remains valid to explain the regulatory influence between proteins. The use of the plaid assumption for the analysis of GIs and PPIs can also provide insights on the network topology and molecular functions, revealing: (1) hubs and core interactions (based on the amount of overlapping interactions), and (2) between- and within-pathway interactions (based on the interactions inside and outside of the overlapping areas). Figure 6 (right) illustrates a plaid model associated with two simple modules with overlapping interactions. These illustrative modules could not be discovered without a plaid assumption.

#### Order-preserving model

An order-preserving module/bicluster is defined by a set of nodes with a preserved relative degree of influence on another set of nodes [22]. Illustrating, given a bicluster (I, J) with I = $$\{x_3,x_5\}$$ and J = $$\{y_2,y_6,y_7\}$$, if $$a_{32}\le a_{36}\le a_{37}$$ then $$a_{52}$$$$\le$$$$a_{56}$$$$\le$$$$a_{57}$$. Assuming that an order-preserving module is observed with two proteins acting as a transcription factors of a set of genes/proteins/metabolites, then these proteins show the same ordering of regulatory influence on the target set of biological entities. Order-preserving modules may contain interactions according to the constant model (as well as modules with shifting and scaling factors [15]), leading to more inclusive solutions associated with larger and less noise-susceptible modules. The order-preserving model is thus critical to accommodate non-fixed yet coherent influence of a node on another set of nodes, tackling the problem of scores’ uncertainty on less-researched regions in the network.

**Fig. 7:**
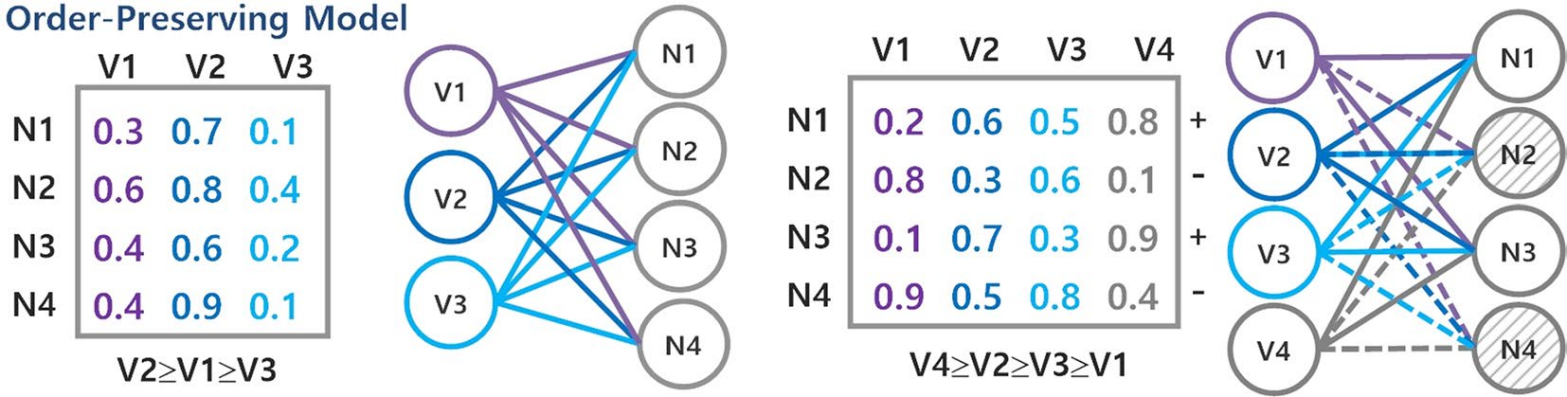
Non-dense biclustering modules: the order-preserving model

An order-preserving coherence with symmetries is often used to model biological settings where the degree of regulations associated with both the activation and repression of groups of genes/proteins/metabolites is preserved. Figure 7 provides illustrative order-preserving modules in the absence and presence of symmetries.

### Handling noisy and missing interactions

An undesirable restriction of existing methods for the discovery of dense modules is that they require almost every node within a module to be connected, thus possibly excluding relevant nodes in the presence of some *missing interactions*. Understandably, meaningful modules with missing interactions are common since the majority of existing biological networks are still largely incomplete.

Pattern-based biclustering is able to recover missing interactions recurring to well-established and efficient postprocessing procedures [44]. These procedures commonly rely on the merging and extension of the discovered modules. Merging is driven by the observation that when two modules share a significant amount of interactions it is probable that their merging composes a larger module still respecting some homogeneity criteria [44]. Extension procedures identify candidate nodes to enlarge a given module (yet still satisfying a certain homogeneity) by changing the minimum support threshold of the pattern-based searches [15]. Furthermore, the scoring scheme of interactions might be prone to *experimental noise* (bias introduced by the applied measurement and preprocessing) and *structural noise* (particularly common in the presence of less researched genes or proteins), not always reflecting the true interactions.

Recent breakthroughs in pattern-based biclustering show the possibility to assign multiple ranges of values on specific interactions (see Fig. 4) to reduce the propensity of excluding interactions due to score deviations. Since pattern mining searches are inherently able to learn from transactions or sequences with an arbitrary number of items, this enables the possibility to assign multiple items to a single element of the mapped matrix. As such, elements with values near a boundary of discretization (or cut-off threshold) can be assigned with two items corresponding to the closest ranges of values. Under this procedure, pattern-based biclustering is able to effectively address different forms of noise based on parameterizable distances for the assignment of additional items.

According to the previous strategies, the level of sparsity and noise of the discovered modules can be parametrically controlled. Illustrating, to strengthen the quality of a given module (reducing its tolerance to noise), the overlapping thresholds for merging procedures can be reduced. Figure 5 provides an illustrative constant module with missing interactions (red dashed lines) and noisy interactions (red continuous lines).

By default, BicNET relies on a merging procedure with an 80 % overlapping threshold (with the computation of similarities pushed into the mining step according to [44]) and on the assignment of multiple items for interactions with scores closer to a boundary of discretization (allocation of 2 items for interactions in a range $a_{ij}\in [c_1,c_2]$ when $\frac{min(c_2-a_{ij},\,a_{ij}-c_1)}{c_2-c_1}<25\, \%$ according to [22]).

### BicNET: efficient biclustering of biological networks

Understandably, the task of biclustering modules with the introduced coherencies is computationally harder than biclustering dense modules (the complexity of biclustering non-dense models is discussed in [15, 22]). Empirical evidence using state-of-the-art biclustering algorithms shows that this task in its current form is only scalable for biological networks up to a few hundreds of nodes [41]. Nevertheless, a key property distinguishing biological networks from gene expression or clinical data is their underlying sparsity. Illustrating, some of the densest PPI and GI networks from well-studied organisms still have a density below 5 % (ratio of interconnected nodes after excluding nodes without interactions) [16].

While traditional biclustering depends on operations over matrices, pattern-based biclustering algorithms are prepared to mine transactions of varying length. This property makes pattern-based biclustering algorithms able to exclude missing interactions from searches and thus surpass memory and efficiency bottlenecks. To understand the impact of this option, given a homogeneous network with *n* nodes, the complexity of traditional biclustering algorithms is bounded by $\Theta (f(n^2))$ (where *f* is the biclustering function), while the target approach is bounded by $\Theta (f(p))$ (where *p* is the number of pairwise interactions) and $p\ll n^2$ for biological network data.

Based on these observations, we propose BicNET (*BiC*lustering Biological *NET*works), a pattern-based biclustering algorithm for the discovery of modules with parameterizable forms of coherency and robustness to noise in biological networks. BicNET relies on the following principles to explore efficiency gains from the analysis of biological networks.

We first propose a new data structure to efficiently preprocess data: an array, where each position (node from a disjoint set in the bipartite graph) has a list of pairs, each pair representing an interaction (corresponding node and the interaction weight). Discretization and itemization procedures are performed by linearly scanning this structure. In this context, the time and memory complexity of these procedures is linear on the number of interactions. Sequential and transactional databases are mapped from this preprocessed data structure without time and memory overhead.

Pattern-based searches commonly rely on bitset vectors due to the need to retrieve not only the frequent patterns but also their supporting transactions in order to compose biclusters. Pattern-based searches for biclustering commonly rely on variants of AprioriTID methods [45] or vertical methods (such as Eclat [46]). However, Apriori-based methods suffer from the costs associated with the generation of a huge number of candidate modules for dense networks or networks with modules of varying size [41], while vertical-based methods rely on expensive memory-and-time costs of intersecting (arbitrarily large) bitsets [47]. These observations can be experimentally tested by parameterizing BicNET with these searches (used for instance in BiModule [23], GenMiner [48] and DeBi [24] biclustering algorithms). For this reason, we rely on the recently proposed F2G miner [47] and on revised implementations of Eclat and Charm miners where diffsets are used to address the bottlenecks of bitsets in order to efficiently discover constant/symmetric/ plaid models, as well as on IndexSpan [22] miner to efficiently discover order-preserving models.

Furthermore, the underlying pattern mining searches of BicNET are dynamically selected based on the properties of the network to optimize their efficiency. Horizontal versus vertical data formats [15] are selected based on the ratio of rows and columns from the mapped matrix. Apriori (candidate generation) versus pattern-growth (tree projection) searches [15] are selected based on the network density (pattern-growth searches are preferable for dense networks). We also push the computation of similarities between all pairs of biclusters (the most expensive postprocessing procedure) into the mining step by checking similarities with distance operators on a compact data structure to store the frequent patterns.

#### Scalability

Additional principles from the research on pattern mining can be used to guarantee the scalability of BicNET.

Multiple parallelization and distribution principles are directly applicable by enhancing the underlying pattern mining searches [49, 50]. Alternatively, data partitioning principles can be considered under certain optimality guarantees [50, 51]. Finally, BicNET can additionally benefit from efficiency gains associated with searches for approximate patterns [22, 50].

### BicNET: incorporating available domain knowledge

As previously discussed, pattern-based biclustering algorithms show the unprecedented ability to efficiently discover exhaustive structures of biclusters with parameterizable coherency and quality. In this context, two valuable synergies can be identified. First, the optimality and flexibility of pattern-based biclustering solutions provide an adequate basis upon which knowledge-driven constraints can be incorporated [39]. Second, the effective use of domain knowledge to guide the underlying pattern mining searches has been largely researched in the context of domain-driven pattern mining [52, 53].

#### Constraint-guided biclustering

In previous work [42], pattern-based biclustering algorithms were extended to optimally explore efficiency gains from constraints with succinct, (anti-)monotone and convertible properties. For this end, F2G and IndexSpan pattern mining searches were revised (and respectively termed F2G-Bonsai and IndexSpanPG [42]) to be able to effectively incorporate and satisfy such constraints for the final task of biclustering expression data. BicNET can be seen as wrapper over existing pattern mining searches, adding new principles to guarantee that they are consistently, robustly and efficiently applied over biological networks. As such, BicNET’s behavior complies with domain-driven pattern mining searches. In fact, domain-driven pattern mining searches, such as F2G-Bonsai and IndexSpanPG, simply provide mechanisms to interpret constraints and guarantee that they are used to guide the pruning of the search space.

**Fig. 8:**
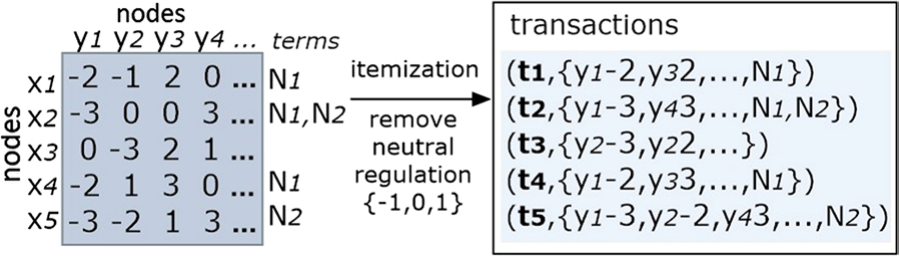
Illustrative symbolic network with annotations

To illustrate some of the meaningful constraints that can be supported in BicNET, consider the biological network provided in Fig. 8. Biological entities are linked through interactions whose strength is either negative {−3, −2} (e.g. inhibition), weak {−1, 0, 1} or positive {2, 3} (e.g. activation). Also, consider the *pattern*$\varphi_B$ of a bicluster with coherency across rows to be the ordered set of expected values on a row in the absence of noise ($\eta _{ij}$ = 0) and plaid effects, $\varphi _B=\cup _{j=1}^{|J|}\{k_j\}$. In this context, let us consider illustrations of meaningful succinct, (anti-)monotone and convertible constraints.

Succinct constraints can be used to remove ranges of uninformative interactions from the network [*remove*(*S*) where $S\subseteq \mathbb {R}^+$ or $S\subseteq \mathcal {L}$]. Illustrating, some labels may not be relevant when mining biological networks with qualitative interactions, while low scores (denoting weak associations) can be promptly disregarded from biological networks with weighted interactions. Despite the structural simplicity of this behavior, this possibility cannot be supported by peer state-of-the-art biclustering algorithms [42].

Succinct constraints can be alternatively used for the discovery of biological entities interacting according to a specific patterns of interest. Illustrating, $\{-2, 2\}\subseteq \varphi _B$ implies an interest on non-dense network modules (interactions without strong weights) to disclose non-trivial regulatory activity, and $min(\varphi _B)= -3\wedge max(\varphi _B)= 3$ implies a focus on modules with interactions delineating strong activation and repression.

Monotone and anti-monotone constraints are key to discover modules with distinct yet coherent regulatory interactions. Illustrating, the non-succinct monotonic constraint *countVal*$(\varphi _B)\ge 3$ implies that at least three different types of interaction’s strengths must be present within a module. Assuming a network with {a,b,c} types of biological interactions, then $|\varphi _B\cap \{a,b\}|\le 1$ is anti-monotone.

Finally, convertible constraints are useful to fix pattern expectations, yet still accommodating deviations from expectations. Illustrating, $avg(\varphi _B)\le 0$ indicates a preference for network modules with negative interactions without a strict exclusion of positive interactions.

#### Integration of external knowledge

BicNET is also able to benefit from network data contexts where nodes can be annotated. These annotations are often retrieved from knowledge repositories, semantic sources and/or literature. Annotations can be either directly derived from the properties of the biological entity (such as functional terms from ontologies) or be implicitly predicted based on the observed interactions (such as topological properties). Illustrating, consider a gene-interaction network where genes are annotated with functional terms from Gene Ontology (GO) [54]. Since a gene can participate in multiple biological processes or, alternatively, its function be yet unknown, genes can have an arbitrary number of functional annotations.

Since pattern mining is able to rely on observations with an arbitrary length, BicNET consistently supports the integrated analysis of network data and annotations. For this aim, annotations are associated with a new dedicated symbol and appended to the respective row in the mapped adjacency matrix (see Fig. 8). Illustrating, consider $T_1$ and $T_2$ terms to be respectively associated with genes $\{x_1,x_3,x_4\}$ and $\{x_3,x_5\}$, an illustrative transactional database for this scenario would be $\{x_1=\{a_{11},\ldots,a_{1m},T_1\},x_2=\{a_{21},\ldots,a_{2m}\},x_3=\{a_{31},\ldots,a_{3m},T_1,T_2\},\ldots\}$. Sequential databases can be composed by appending terms either at the end or the beginning of each sequence.

Given these enriched databases, pattern mining can then be applied with succinct, (anti-)monotone and convertible constraints. Succinct constraints can be incorporated to guarantee the inclusion of certain terms (such as $\varphi _B\cap \{T_1,T_2\} \ne0$). (Anti-)monotone convertible constraints can be, alternatively incorporated to guarantee that, for instance, a bicluster is functionally consistent, meaning that it can be mapped to a single annotation. The $|\varphi _B\cap \{T_1,T_2\}|\le 1$ constraint is anti-monotone and satisfies the convertible condition: if $\varphi _B$ satisfies the constraint, the $\varphi _B$ suffixes also satisfy the constraint.

### Benefits of BicNET against its peers

**Fig. 9:**
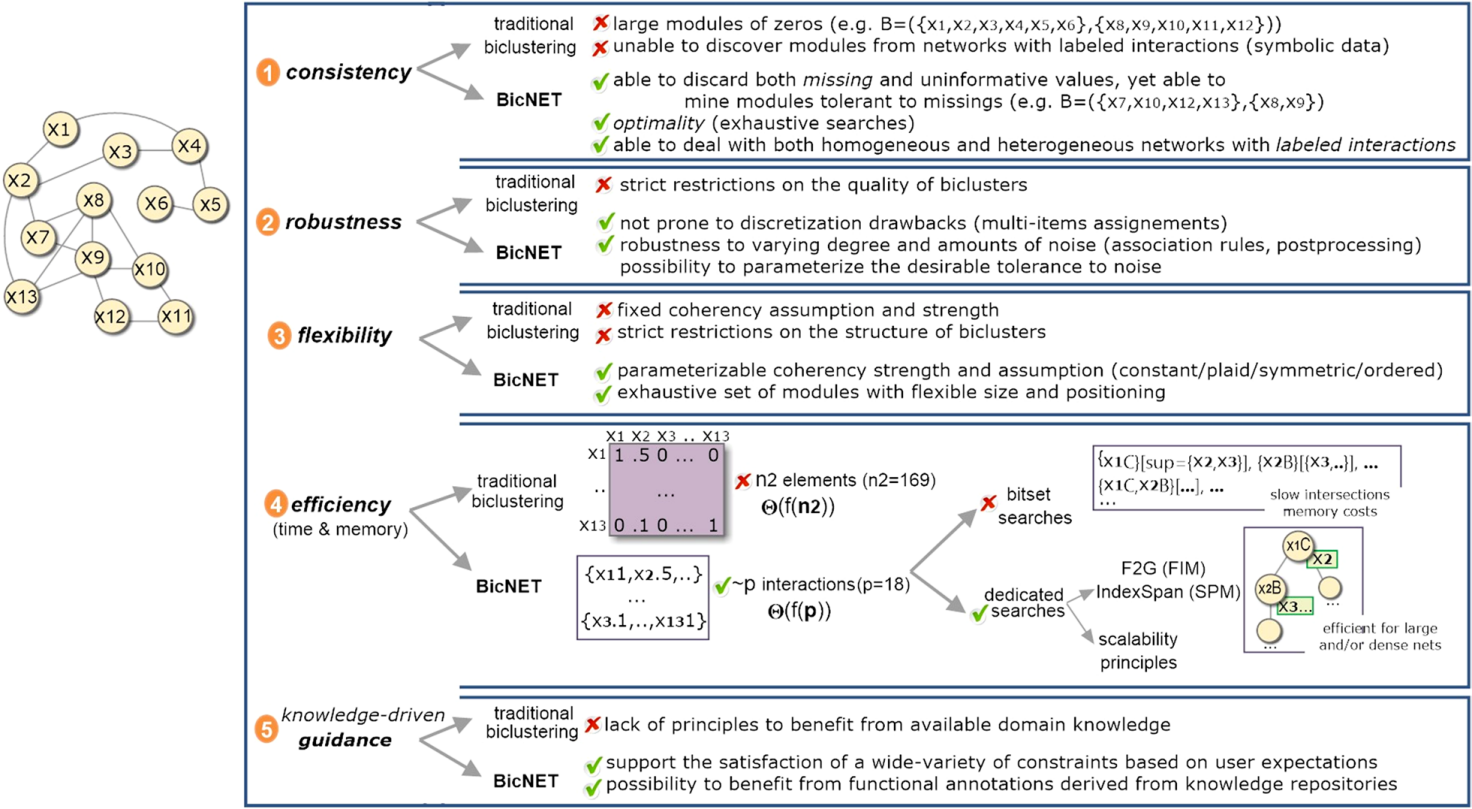
Tackling the existing limitations with BicNET: *1* addressing inconsistencies and guarantee the applicability towards different types of network; *2* enabling for the first time the discovery of modules with varying coherency criteria; *3* guaranteeing the robustness of the searches and the possibility to parameterize the desirable quality of the modules; *4* surpassing efficiency bottlenecks of state-of-the-art and peer pattern-based biclustering algorithms; and *5)* benefiting from the guidance of available background knowledge

This section introduced respectively principles to guarantee the *consistency*, *flexibility*, *robustness* and *efficiency* of BicNET, as well as its ability to benefit from guidance in the presence of domain knowledge. Figure 9 illustrates the positioning of BicNET on each one of these qualities against alternative state-of-the-art biclustering algorithms.

Additional opportunities of BicNET include the:

- possibility to analyze not only biological networks but also sparse biological matrices, such as expression data (where non-differential expression is removed) and genome structural variations (where entries without mutations or single-nucleotide polymorphisms are ignored);
- easy extension of BicNET for the discovery of discriminative modules for labeled or class-conditional biological networks by parameterizing BicNET with discriminative pattern mining searches [55, 56];
- incorporation of statistical principles from pattern mining research [57–59] to assess the statistical significance of modules given by pattern-based biclusters, thus guaranteeing the absence of false positive discoveries [18].

## BicNET: algorithmic aspects

The algorithmic basis of BicNET is described in Algorithm 1. BicNET’s behavior can be synthesized in three major steps: mapping, mining and postprocessing. First, the input network is mapped into one or more minimal (sparse) adjacency matrices, being the number of generated matrices given by $$\left( {\begin{array}{c}max(\kappa ,2)\\ 2\end{array}}\right)$$ where $\kappa$ is the number of distinct types of nodes from the inputted network. For example, 6 adjacency matrices would be generated for a biological network capturing interactions between genes, protein, protein complexes and metabolites. Each adjacency matrix is efficiently represented using an array of lists of pairs, where each position in the array stores both the index/ID of the nodes interacting with a given node as well as the values for those interactions. If the inputted interactions are labeled or unweighted, BicNET proceeds directly with the mining step. If the inputted interactions have real-valued weights, they are discretized (after proper normalization and exclusion of outliers) under a given coherency strength determining the length of the alphabet for discretization. Multiple items can be assigned (according to "Handling noisy and missing interactions" section) to mitigate the drawbacks associated with the discretization needs. Due to the assignment of multiple items, each list from the array may have duplicated indexes/IDs. In the absence of a prespecified coherency strength, BicNET iteratively discretizes the adjacency matrices using several alphabets. The modules discovered under each coherency strength are jointly postprocessed.

**Fig. 10:**
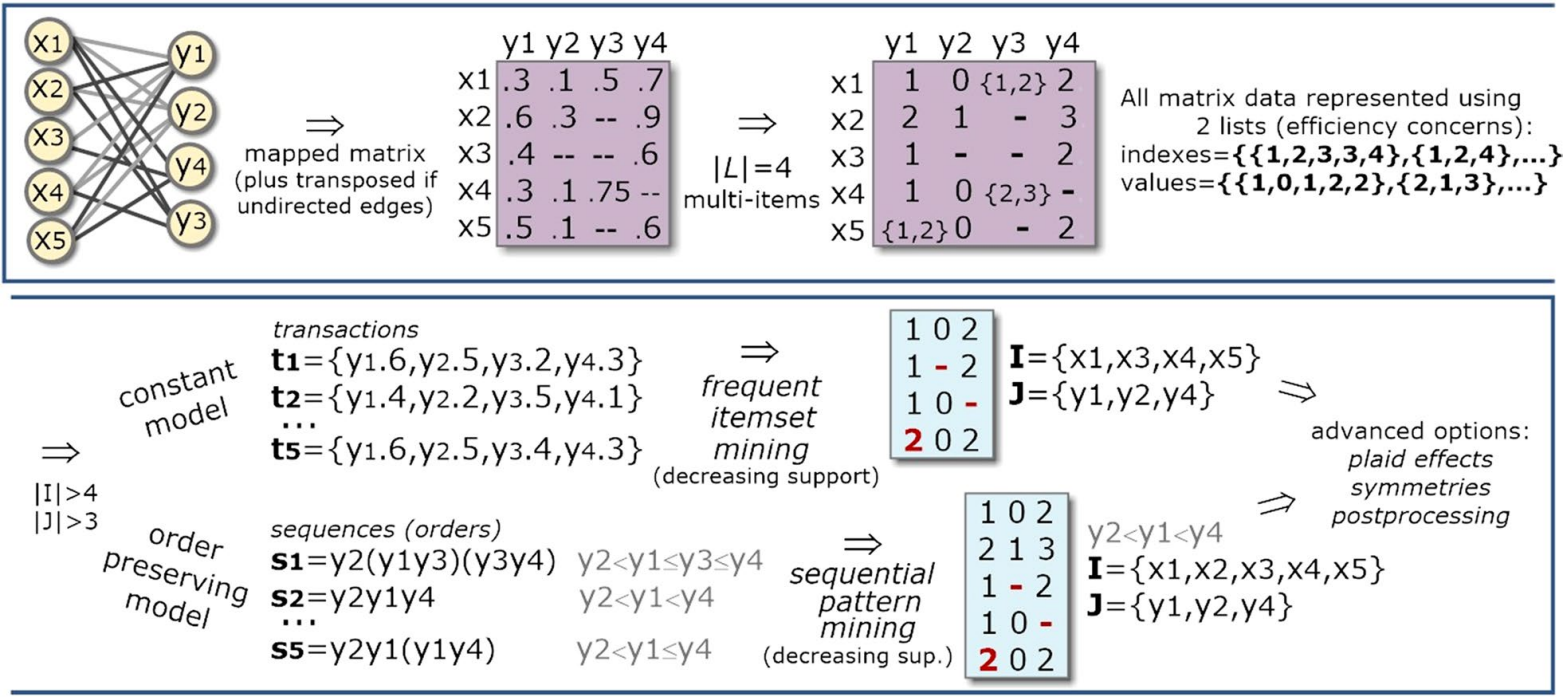
Simplified illustration of BicNET behavior: efficient storage of multi-item discrete adjacency matrices mapped from network data; iterative application of distinct pattern mining searches with decreasing support for the discovery of modules with varying coherency criteria; and postprocessing of the discovered modules

Second, transactional and sequential databases are mapped from the previous data structures and pattern mining searches iteratively applied (see Fig. 3). Transactional databases are used for the discovery of constant/symmetric/plaid modules, while sequential databases (where discretization is optional) are considered for the discovery of order-preserving modules. In the context of transactional databases, the values of each pair (node index/ID, value) are concatenated to generate transactions of items. Given a transactional database, frequent itemset mining (for the discovery of noise-intolerant constant biclusters [18]) or association rule mining (for noise-tolerant constant biclusters [21]) are iteratively applied with a decreasing support until a high number of biclusters (or coverage of the inputted network of interactions) is achieved. In the context of sequential databases, the node indexes/IDs that interact with a given node are sorted according to the associated values to compose sequences of indexes. Given a sequential database, sequential pattern mining is then iteratively applied with a decreasing support for the discovery of order-preserving biclusters. Figure 10 provides a simplified illustration of these major steps for the task of discovering constant and order-preserving modules.

**Fig. 11:**
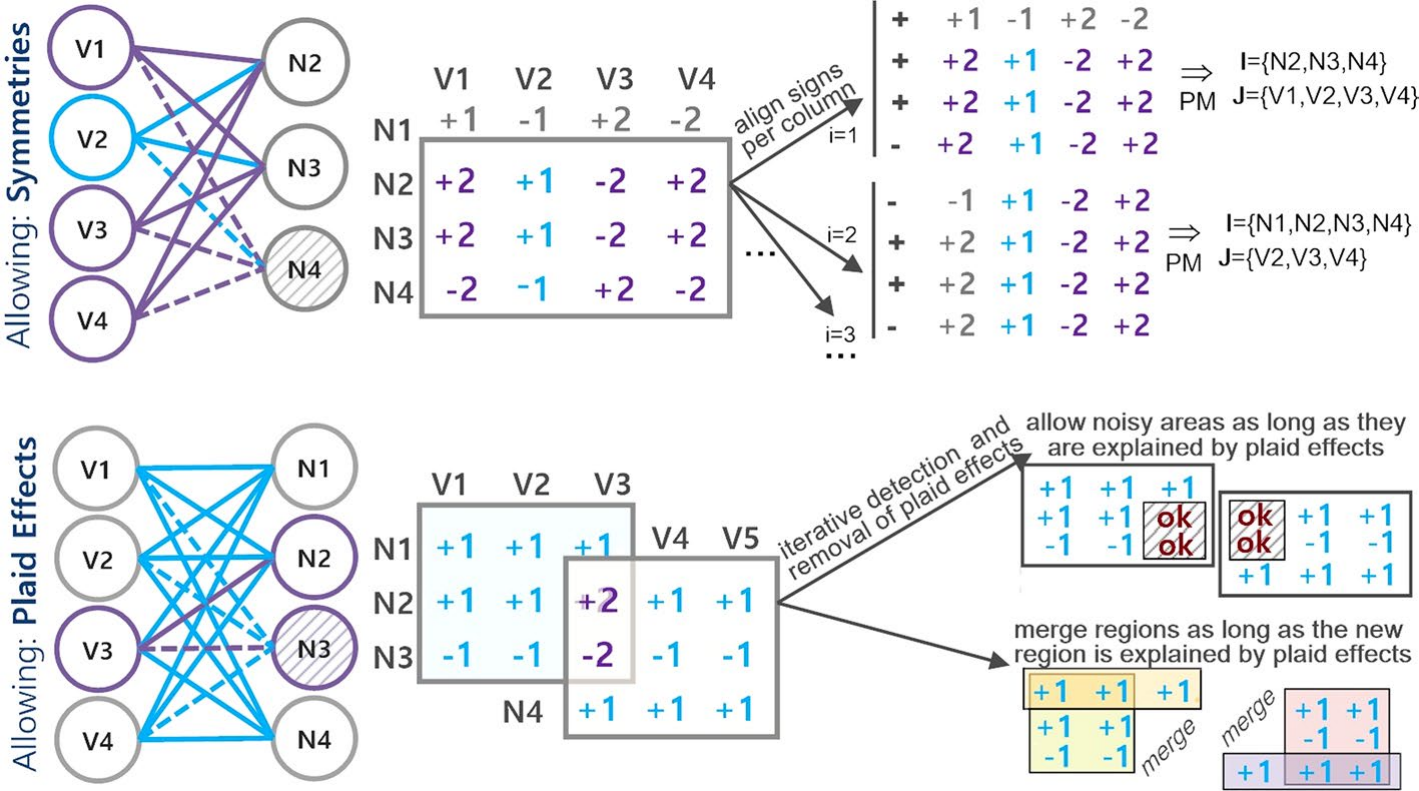
Advanced aspects of BicNET: *1* allowing symmetries within the discovered modules through iterative sign adjustments to model biological entities simultaneously involved in up- and down-regulatory interactions, and *2* allowing plaid effects through the guided inclusion of new interactions explained by cumulative contributions to model biological entities involved in multiple biological processes (commonly associated with overlapping regions or hub-nodes within a network)

Understandably, additional strategies need to be present to discover modules with more intricate coherency aspects. As introduced, modules with symmetric effects are essential to model biological entities that coherently establish both upstream and downstream regulatory interactions with an additional set of nodes, while modules with plaid effects are essential to model cumulative contributions in the interactions from biological entities participating in more than one module/putative biological process. For the discovery of modules with symmetries, BicNET iteratively performs sign corrections on the mapped data, executing the mining step for each adjusted dataset (see Fig. 9). Pruning principles are made available (according to [15]) to guarantee the efficiency of these searches. For the discovery of modules wit plaid effects, three principles are considered. Modules with high tolerance to noise are discovered by performing association rule mining with low confidence thresholds (as described in [21]), and the nodes with noisy interactions within each module are tested in order to check whether their interactions are explained by cumulative contributions. The inclusion of regions explained by plaid effects and the removal of noisy interactions is performed iteratively according to the BiP algorithm [21] in order to be able to deal with an arbitrary-high number of cumulative contributions. BiP is formally described in Appendix. Figure 11 provides a simplified illustration of how BicNET is able to accommodate symmetric and plaid effects.
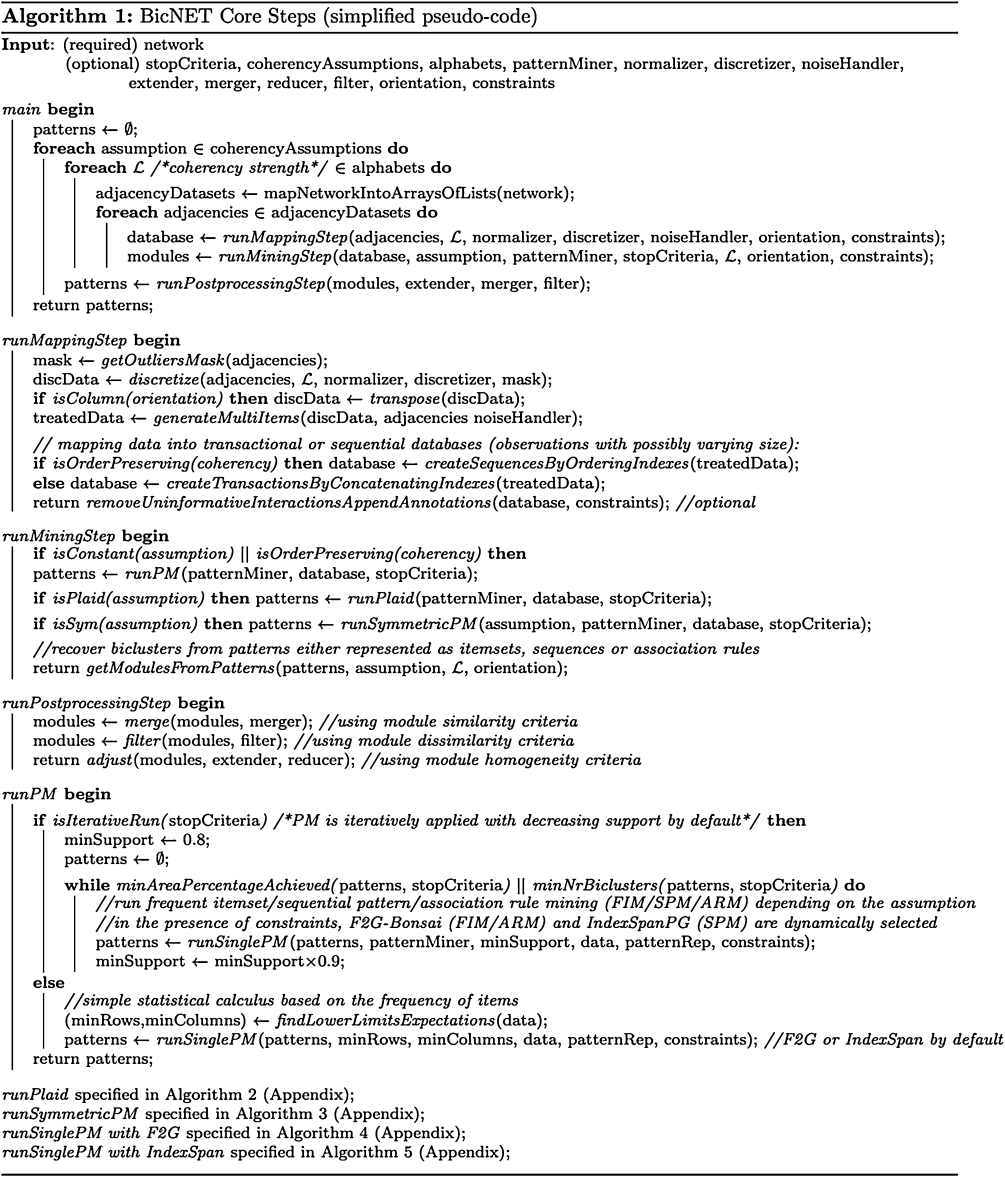

Domain knowledge and user expectations can be declaratively specified as a set constraints and inputted as a parameter to BicNET. For this aim, BicNET simply replaces the underlying pattern mining searches by F2G-Bonsai (for the constant/symmetric/plaid model) or IndexSpanPG (for the order-preserving model) [42].

Third and finally, postprocessing procedures to merge, filter, extend or reduce modules are applied according to the principles respectively introduced in "Handling noisy and missing interactions" and "BicNET: efficient biclustering of biological networks" sections.

### Computational complexity

The computational complexity of BicNET is bounded by the pattern mining task and computation of similarities among biclusters. For this analysis, we discuss the major computational bottlenecks associated with each one of the three introduced steps. The discretization (including outlier detection and normalization) and noise correction procedures (for the assignment of multiple items) within the *mapping* step are linear on the size of the matrix, $$\Theta (p)$$, where *p* is the number of interactions and typically $$p\ll n^2$$. To dynamically select an adequate discretization procedure, distribution fitting tests and parameter estimations^3^ are performed in $$\Theta (p)$$. The complexity of the *mining step* depends on three factors: the complexity of the pattern miner and the amount of iterations need for the discovery of modules with varying coherency assumptions. The cost of the pattern mining task depends essentially on the number and size of transactions/sequences (essentially defined by the size and sparsity of the inputted network), selected mining procedures (FIM, SPM or association/sequential rules defined by the desired coherency assumption) and respective algorithmic implementations, the frequency distribution of items (essentially defined by the target coherency strength), the selected pattern representation (closed by default), and the presence of scalability enhancements (listed throughout "BicNET: efficient biclustering of biological networks" section). Empirical evidence shows that the complexity of the mining step, when iteratively applied with a decreasing support threshold, is bounded by the search with lowest support. A detailed analysis of the complexity of the pattern mining task has been attempted in literature [60] and it is out of the scope of this paper. Let $$\Theta (\wp )$$ be the complexity of the pattern mining task. For the discovery of symmetric and plaid effects, the previous mining procedure is iteratively applied, being the final search bounded by $$\Theta (d$$$$\times$$$$\wp )$$, where $$d\approx {n \atopwithdelims ()2}$$. Finally, the complexity of the *postprocessing* step depends essentially on two factors: (1) the complexity of computing similarities among biclusters to merge and filter modules (bounded by $$\Theta ({k \atopwithdelims ()k/2}\bar{r}\bar{s})$$ based on [15], where *k* is the number of modules and $$\bar{r}\bar{s}$$ is the average number of interactions per module), and (2) the complexity of extending and reducing modules (bounded by $$k'(\bar{r}n+n\bar{s})$$, where $$k'$$ is the number of biclusters after merging and filtering). Summing up, the complexity of BicNET is bounded by $$\Theta (d\wp +{k \atopwithdelims ()k/2}\bar{r}\bar{s}+k'(\bar{r}n+n\bar{s}))$$, which for large-scale networks (where typically *k*$$\gg$$$$k'$$) is approximately given $$\Theta (d\wp$$ + $${k \atopwithdelims ()k/2}\bar{r}\bar{s})$$.

### Default and dynamic parameterizations

As BicNET makes available a high number of options and thus fine tunable parameters, there is the need to guarantee that it provides a robust and friendly environment to be used by users without expertise in network module discovery and pattern-based biclustering.

For this aim, BicNET makes available: (1) default parameterizations (data-independent setting) and (2) dynamic parameterizations based on the properties of the input dataset (data-dependent setting). Default parameterizations include: (1) zero-mean row-oriented normalization followed by overall Gaussian discretization with *n*/4 items for order-preserving coherencies (for an adequate trade-off of precedences vs. co-occurrences) and a number of items in the set $\{3,5,7\}$ for the remaining coherencies; (2) iterative discovery of modules with distinct coherencies (dense, constant, symmetric, plaid and order-preserving); (3) F2G search for closed FIM and association rule mining, and IndexSpan search for SPM; (4) multi-items assignment (according to criteria introduced in section “Handling noisy and missing interactions”); (5) merging procedure with the computation of Jaccard-based similarities pushed into the mining step and an 80 % overlapping threshold; (6) filtering procedure for biclusters without statistical significance (according to [44]) and a 70 % Jaccard-based similarity against a larger bicluster; and (7) no extension or reduction procedures. For the default setting, BicNET iteratively decreases the support threshold by 10 % (starting with $\theta$ = 80 %) until the output solution discovers 50 dissimilar modules or a minimum coverage of 10 % of the elements in the inputted network interactions.

The dynamic parameterizations differ with regards to the following aspects: (1) the fit of different distributions are tested to select adequate normalization and discretization procedures, (2) the size and sparsity of the biological network are used to affect the pattern mining search (according to [18]), and (3) data partitioning procedures are considered for large-scale networks with over 100 million of interactions for dense and constant module discovery and 1 million of interactions for the discovery of modules with alternative coherency assumptions.

### Software

BicNET is provided within both graphical and programmatic interfaces^4^ to offer a supportive environment for the analysis of biological networks. BicNET supports the loading of input data and the exportation of results according to a wide-variety of formats.

**Fig. 12:**
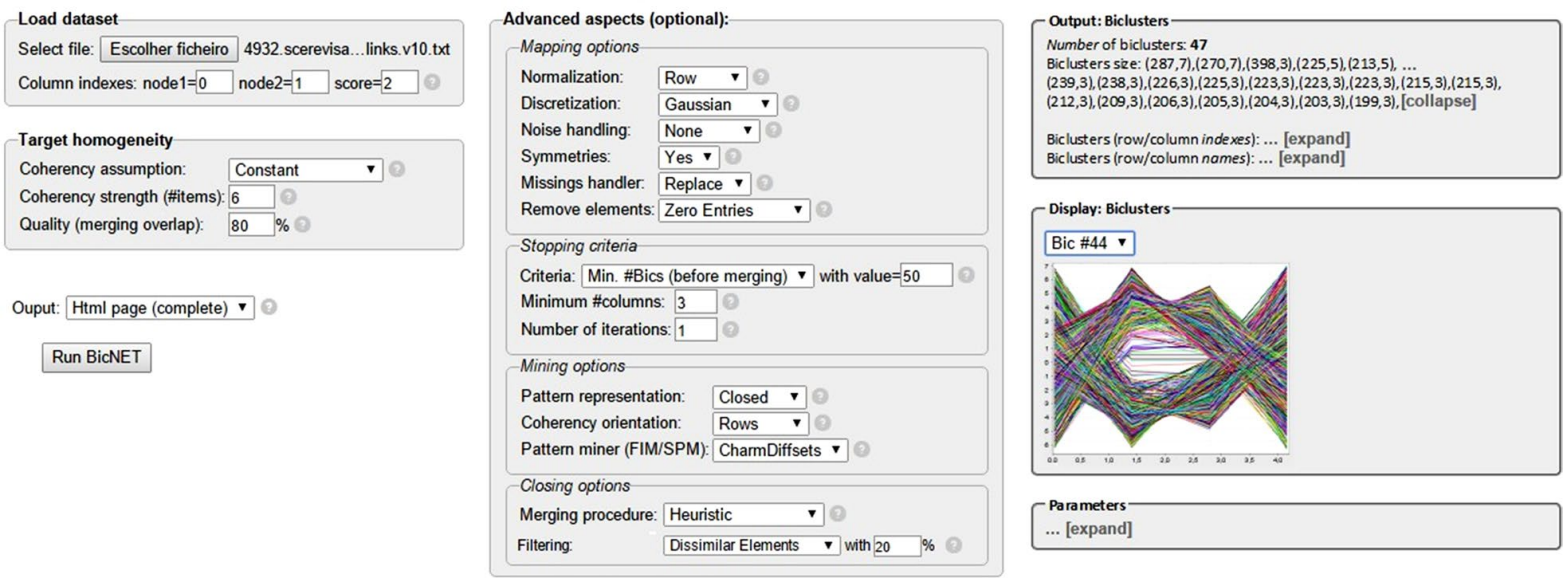
BicNET graphical interface for sound parameterizations and visual analyzes of results

The web-based *graphical interface* of BicNET can be used to soundly parameterize the searches and visualize the outputs. Figure 12 provides an illustrative snapshot of the graphical interface. Soundness is guaranteed by disabling options when certain parameters are selected, providing form checks and adequately displaying possible causes of error (such as data inconsistencies or timeout alerts for extremely heavy requests). This interface is compatible with all browsers and the privacy of the requests is guaranteed. Upon running BicNET, when the stopping criteria is met, a message of success is displayed, enabling the presentation of the output. Both textual and graphical presentations of the discovered biclusters are provided. Biclusters can be sorted, filtered and exported to be visualized by alternative software or stored in knowledge bases. These outputs can be displayed on the website or via email.

Alternatively, BicNET is made available through a *programmatic interface* based on a Java API with the respective source code and accompanying documentation. This interface can be used to: extend pattern-based biclustering algorithms for alternative tasks, such as classification and indexation, and easily adapt its behavior in the presence of biological networks with very specific regularities. Illustrative cases are provided in the webpage of the authors.

## Results and discussion

Results are organized as follows. First, we describe the selected data settings, metrics and algorithms. Second, we compare the performance of BicNET against state-of-the-art algorithms for biclustering and network module discovery, using synthetic networks with varying properties. Finally, we use BicNET for the analysis of large-scale PPI and GI networks to show the relevance of discovering modules with varying forms of coherency and parameterizable levels of noise and sparsity. BicNET is implemented in Java (JVM v1.6.0-24). Experiments were run using an Intel Core i5 2.30GHz with 6GB of RAM.

### Experimental settings

#### Synthetic data

Networks with planted biclusters were generated respecting the commonly observed topological properties of biological networks [41]. For this end, the following key variables were varied:

- Size of networks: number of nodes and density;
- Distribution of the weight of interactions for real-valued networks (Uniform or Gaussian assignment of positive and negative ranges of values) and of labels for symbolic networks;
- Number, size (Uniform distribution on the number of nodes to plant biclusters with dissimilar size), overlapping degree, and shape (imbalance on the distribution of nodes per disjoint set) of modules;
- Modules’ coherency: dense, constant, symmetric, plaid (according to [21]) and order-preserving assumptions, with the respective 1.2, 1, 1.2, 1.1 and 1.5 scale adjustments to the expected size (to guarantee their statistical significance as the different coherency assumptions impact the probability of module to unexpectedly occur by chance);
- Planted degree of noisy and missing interactions (from 0 to 20 %).

**Table 1:** Default synthetic data benchmarks for network data analyzes

	Network nodes (10 % density)					Network density (2000 nodes)			
	200	500	1000	2000	10,000	1 %	5 %	10 %	25 %
Nr. of hidden modules	5	10	15	20	30	3	5	10	20
Nr. of nodes per module	[20, 30]	[30, 40]	[40, 50]	[50, 70]	[100, 140]	[50, 70]	[50, 70]	[50, 70]	[50, 70]
% interactions in modules	19.5	12.2	7.6	4.5	1.1	22.5	9.0	4.5	2.3

Table 1 summarizes the default data settings for some of these variables when assuming that the generated network is homogeneous. The generation of heterogeneous networks is also made available through the specification of the size of each disjoint set of nodes and pairwise density between the sets of distinct types of nodes. For a sound evaluation of the target algorithms, 30 data instances were generated for each data setting.

#### Real data

We used four biological networks: two distinct GI networks for yeast according to DryGIN [19] and STRING v10 [16] databases, and two licensed PPIs from human and *Escherichia coli* organisms from STRING v10 database [16]. The scores in these networks reveal the expected strength of influence/physical interaction between genes/proteins. DryGIN networks are inferred from experimental data, while STRING networks are primarily inferred from literature and knowledge bases. Table 2 shows some basic statistics of the selected networks.

**Table 2:** Biological networks used to assess the relevance and efficiency of BicNET

Type	Organism	$\sharp$Nodes	$\sharp$Interactions	Density (%)	Notes
GI	Yeast	4455	1,91,309	1.0	Links (65 % negative) from double-mutant arrays [19]
GI	Yeast	6314	4,23,335	1.1	Known and predicted associations benchmarked from multiple data sources and text mining, and combined through an integrative score [16]
PPI	*E. Coli*	8428	32,93,416	4.6	
PPI	Human	19,247	85,48,002	2.3	

#### Performance metrics

Given the set of planted modules $\mathcal {H}$ in a synthetic network, the accuracy of the retrieved modules $\mathcal {B}$ is here given by two match scores [(see (1)]: $MS(\mathcal {B},\mathcal {H})$ defining the extent to what found biclusters match with hidden biclusters (completeness/coverage), and $MS(\mathcal {H},\mathcal {B})$ reflecting how well the hidden biclusters are recovered (precision). The presented scores in this work are the average matches collected from 30 instantiations of synthetic networks. These accuracy views surpass the incompleteness of the Jaccard matching scores (only focused on one of the two subsets of nodes at a time [61]) and the loose matching criteria of relative non-intersecting area (RNAI) [62]. Efficiency, statistical and biological significance are used to complement this analysis.

1 $$\begin{aligned} {\mathbf {MS}} ({\mathcal {B}},{\mathcal {H}})=\frac{1}{|\mathcal {B}|} \Sigma _{(I_1,\,J_1)\in {\mathcal {B}}}max_{(I_2,\,J_2)\in {\mathcal {H}}} \sqrt{\frac{|I_1\cap I_2|}{|I_1\cup I_2|}\frac{|J_1\cap J_2|}{|J_1\cup J_2|}} , \end{aligned}$$

#### Introductory notes on tools for network data analysis

As surveyed, a wide diversity of algorithms and tools have been proposed for the modular analysis of biological networks. For this end, three major options have been considered: (1) exhaustive clustering (discovery of sets of nodes *C* such that $\cup _{k}C_k= X \wedge \cap _{k}C_k =\emptyset$) using different algorithms; (2) non-exhaustive clustering with the allowance of overlapping nodes between clusters ($\cup _{k}C_k\subseteq X$); and (3) biclustering (discovery of bi-sets of nodes (*I*, *J*) coherently related). Table 3 provides a compact view on the differences between the solutions gathered by the different techniques, disclosing their intrinsic limitations for the discovery of coherent modules within the target synthetic and biological networks. For this end, kMeans, affinity-propagation and spectral clustering algorithms [63] for weighted networks were tested using MEDUSA software [64], CPMw (clique percolation method for weigthed networks) algorithm [65] using CFinder software was applied for non-exhaustive clustering, and traditional algorithms for biclustering dense network modules (based on the discovery of hypercliques from unweighted and/or weighted networks [6, 8, 11, 12]) were applied using BicNET software.

This analysis highlights some limitations of clustering algorithms, including their sensitivity to a (prespecified or estimated) number of clusters, efficiency bottlenecks for large-scale networks, and solutions with a large number of clusters/modules without statistical and/or biological significance. Also, the set of modules discovered with clustering algorithms strongly differs from biclustering-based modules since the similarity criteria placed by state-of-the-art clustering techniques disregards the coherency of local interactions within the module. Instead, the similarity criteria is primarily driven by the global interactions that each node establish with all of the remaining nodes in the network and by additional topological information pertaining to each node. Based on these observations, the conducted experimental analyzes in this section will primarily concern assessing the performance of BicNET against alternative biclustering algorithms.

**Table 3:** Comparison of widely-used tasks for modular analysis of networks using the introduced synthetic and real datasets

Approach	Method	Solution aspects and concerns	Efficiency
Clustering (exhaustive and non-overlapping node coverage)	k-Means	Majority of clusters show loose connectedness; High variation on the size of modules (1-to-3 clusters covering almost all nodes and the remaining clusters being statistically non-significant [66])	Efficiency problems for networks with >100.000 interactions
	Spectral	Able to isolate modules where the degree of connectedness is approximately constant per module; Only a small subset of clusters is relevant (medium-to-high degree of connectedness)	Medusa implementation only scales for networks with <10.000 interactions
	Affinity propagation	The clusters collected from (small samples of) the target biological networks show a generalized lack of biological relevance	Time and memory bottlenecks for small nets (<1000 interactions)
Clustering (non-exhaustive and possibly overlapping node coverage)	CPMw (weighted *k*-clique percolation)	Intolerance to noise; Intractably large solutions (explosion of similar clusters) with strict coherency criterion (*k*-clique); Dependence on parameters (e.g. *k*, intensity level)	Only scales for nets with <5000 nodes (5–10 % density). Bottlenecks for the target biological data even when removing >95 % interactions
Biclustering (bi-sets of nodes)	Hypercliques (unweighted)	Intolerant to missing interactions; Large number of highly similar modules; Dense coherency only	BicNET implementation efficient for large networks (>10000 nodes) with density up to 25 %
	Hypercliques (differential)	Intolerant to noise and the prone item-boundaries problem during the selection of differential weights; Dense coherency only	BicNET implementation scales for large dense networks
	BicNET (dense assumption)	Focus on dissimilar modules robust to noise and missings, with possibly distinct forms of coherency strength (|*L*| $\in${1,2,3,5})	Efficiency bounded by the search for unweigthed hypercliques (|*L*|=1)

#### Algorithms for comparisons

For the purpose of establishing fair comparisons, we select 7 state-of-the-art biclustering algorithms that, similarly to BicNET, are prepared to find biclusters with non-dense coherencies^5^: FABIA^6^ [67], ISA [69], xMotifs [70] and Cheng and Church [71] (all able to discover variants of the introduced constant model); OPSM [72] and OP-Clustering [43] (able to discover order-preserving models); and SAMBA [20] (inherently prepared to discover dense biclusters). The number of seeds for FABIA and ISA was set to 10 and the number of iterations for OPSM was varied from 10 to 100. The remaining parameters of the selected methods were set by default.

### Results on synthetic data

**Fig. 13:**
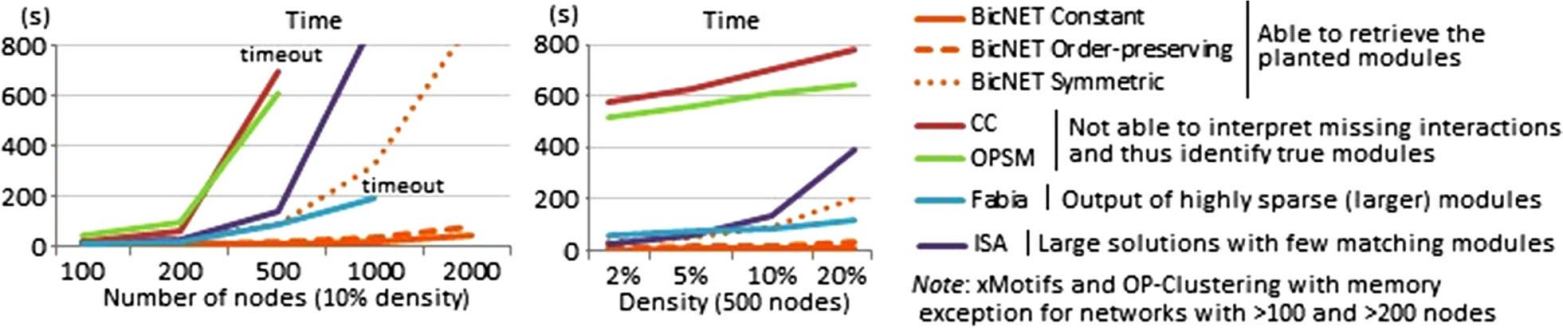
Efficiency of biclustering algorithms able to discover non-dense modules for synthetic networks with varying size and density

In Fig. 13, we compare the efficiency of BicNET with state-of-the-art biclustering algorithms with non-dense coherency criteria for the analysis of networks with varying size and density and planted modules following a constant coherency assumption.

Three major observations can be retrieved. First, BicNET shows heightened efficiency levels, constrasting with peer biclustering algorithms. Understandably, as most of the remaining algorithms are only prepared to analyze (non-sparse) matrices, they show efficiency bottlenecks for even small networks. Second, the majority is not able to accurately recover the planted modules as they cannot interpret missing interactions. Third, although SAMBA [20] and some pattern-based biclustering algorithms, such as BiMax and DECOB [8, 12], are able to discover dense models efficiently, they are not prepared to discover modules with alternative coherence criteria.

**Fig. 14:**
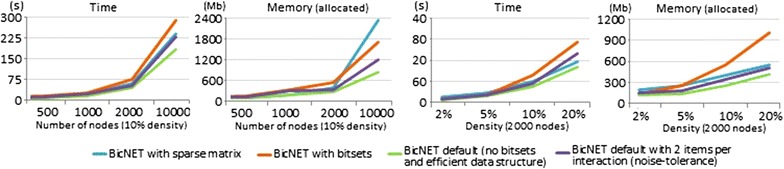
Efficiency gains of BicNET when using sparse data structures, pattern mining searches providing robust alternatives to bitset vectors, and noise handlers

Figure 14 zooms-in the performance of BicNET, quantifying the efficiency gains in terms of memory and time from using adequate data structures (replacing the need to use matrices) and searches (replacing the need to rely on bitset vectors). It also shows that the costs of assigning multiple symbols per interaction are moderate, despite resulting in an increased network density.

**Fig. 15:**
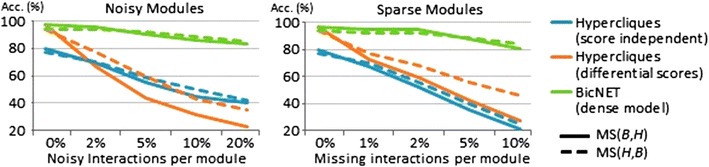
Accuracy of BicNET against pattern-based biclustering algorithms on networks for the discovery of dense modules with varying degree of noisy and missing interactions (networks with 2000 nodes and 10 % density)

Figure 15 compares the performance of BicNET with peer algorithms for discovering dense network modules (hypercliques) in the presence of noisy and missing interactions. This analysis clearly shows that existing pattern-based searches for hypercliques have no tolerance to errors since their accuracy rapidly degrades for an increased number of planted noisy/missing interactions. Thus, they are not able to deal with the natural incompleteness and scoring uncertainty associated with biological networks. On the other hand, the observed accuracy levels of BicNET demonstrate its robustness to noise (validating the importance of assigning multiple ranges of weights for some interactions) and to missing interactions (showing the effectiveness of BicNET’s postprocessing procedures).

**Fig. 16:**
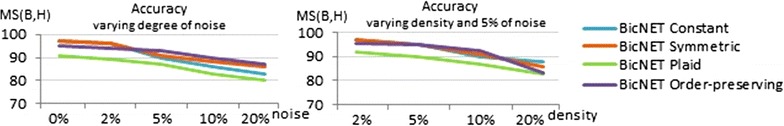
Assessment of BicNET’s ability to recover planted modules with constant, symmetric, plaid and order-preserving coherencies from noisy networks (networks with 2000 nodes according to Table 1)

Finally, Fig. 16 shows that, even in the presence of medium-to-high levels of noise, BicNET can be effectively applied for the discovery of modules with distinct coherencies. All of the target coherencies are associated with searches showing high levels of accuracy, with the plaid model being slightly worse than its peers due to the inherent harder nature of this task when multiple modules overlap according to a complex schema. Additionally, order-preserving models have higher propensity to define modules with false positive nodes for dense networks due to the higher probability of background values to respect this coherency.

### Results on real data

Results gathered from the application of BicNET over real biological networks are provided in three parts. First, we show basic statistics that motivate the relevance of using BicNET against peer algorithms. Second, we explore the biological relevance of the retrieved modules when considering varying levels of tolerance to noise and different forms of coherency. Finally, we make use of some of the meaningful constraints provided in "BicNET: incorporating available domain knowledge" section in order to discover less-trivial modules (such as modules characterized by the presence of plaid effects, flexible constant patterns or symmetries), and provide a brief analysis of their enriched terms and transcription factors.

The biological significance of the retrieved modules from real data is here computed by assessing the over-representation of Gene Ontology (GO) terms with an hypergeometric test using GOrilla [73]. A module is significant when its genes or proteins show enrichment for one or more of the “biological process” terms by having a (Bonferroni corrected) *p* value below 0.01.

**Fig. 17:**
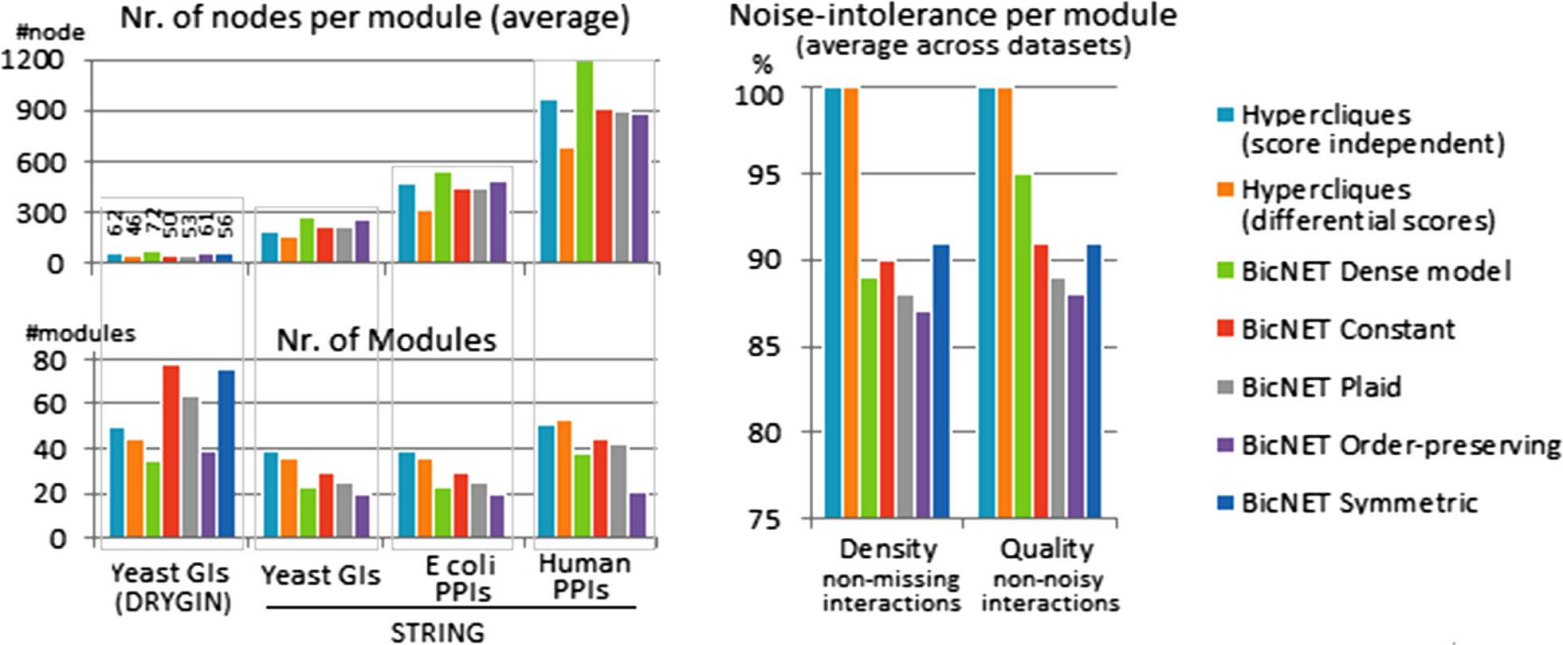
Properties of BicNET solutions against hypercliques discovered in GI and PPI networks (described in Table 2) when considering varying coherency criteria

Figure 17 shows some of the properties of BicNET solutions for the four biological networks described in Table 2. In particular, 97 % of the BicNET’s modules discovered in DRYGIN’s yeast GIs were significantly enriched, while all the BicNET’s modules discovered in STRING’s yeast GIs were significantly enriched. BicNET is able to discover the largest number of (non-similar and statistically significant) biclusters. The analysis of the enriched terms for these modules (see Tables 4, 6) against the significant terms found in other biclustering solutions supports the completeness of BicNET’s solutions, as well as their exclusivity and relevance since the majority of the enriched modules were not discovered by peer algorithms (see Table 5). The biological significance of peer biclustering algorithms focused on dense regions is further hampered by noise and discretization errors (in accordance with Fig. 17). Alternative biclustering algorithms able to discover non-dense regions were not able to scale. The subsequent analyzes (Tables 4, 5, 6, 7) provide further empirical evidence for the relevance, completeness and exclusivity of BicNET solutions.

#### Modules with varying coherency

**Fig. 18:**
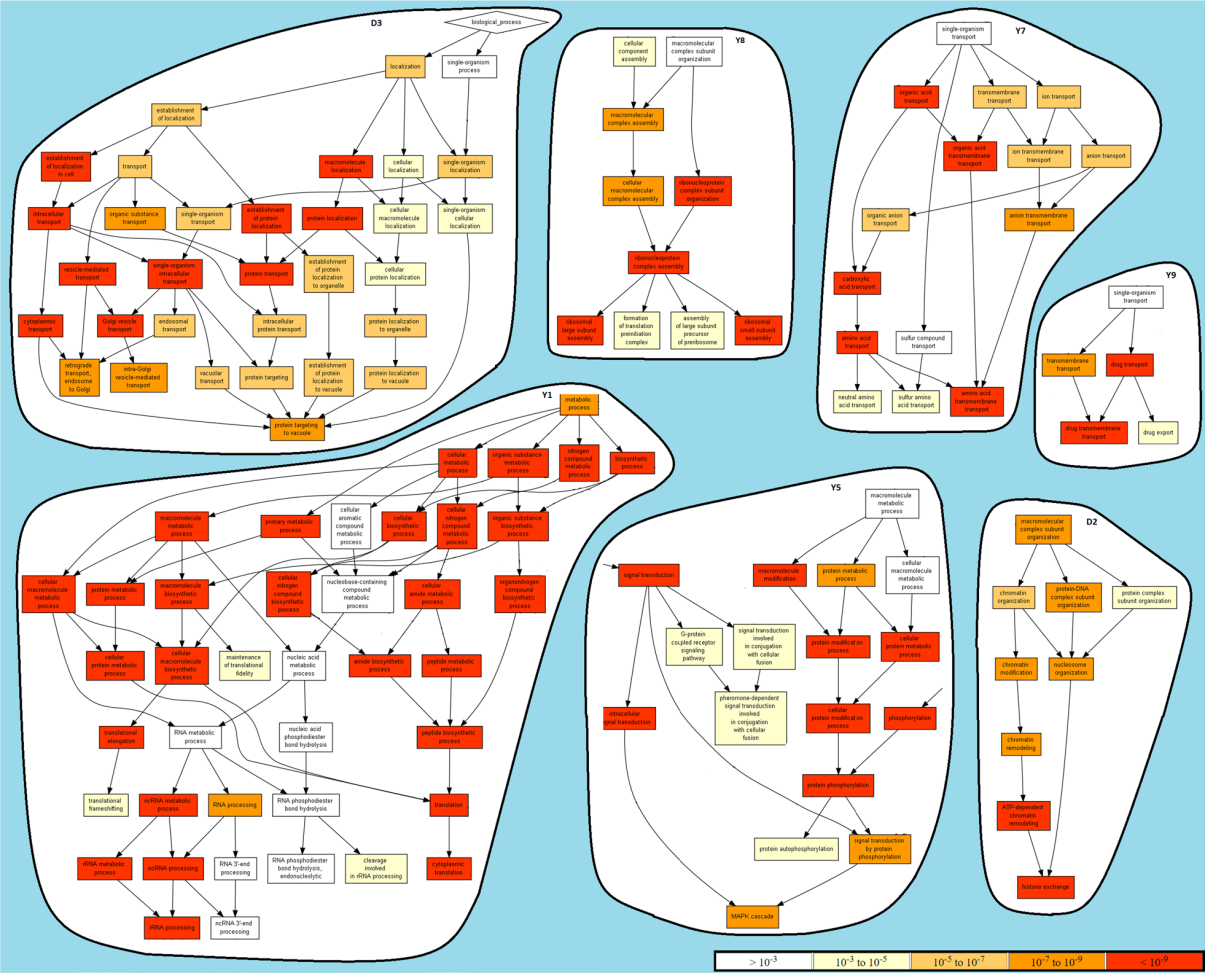
Taxonomy of enriched terms for BicNET’s modules from yeast GIs (on STRING and DryGIN networks)

**Fig. 19:**
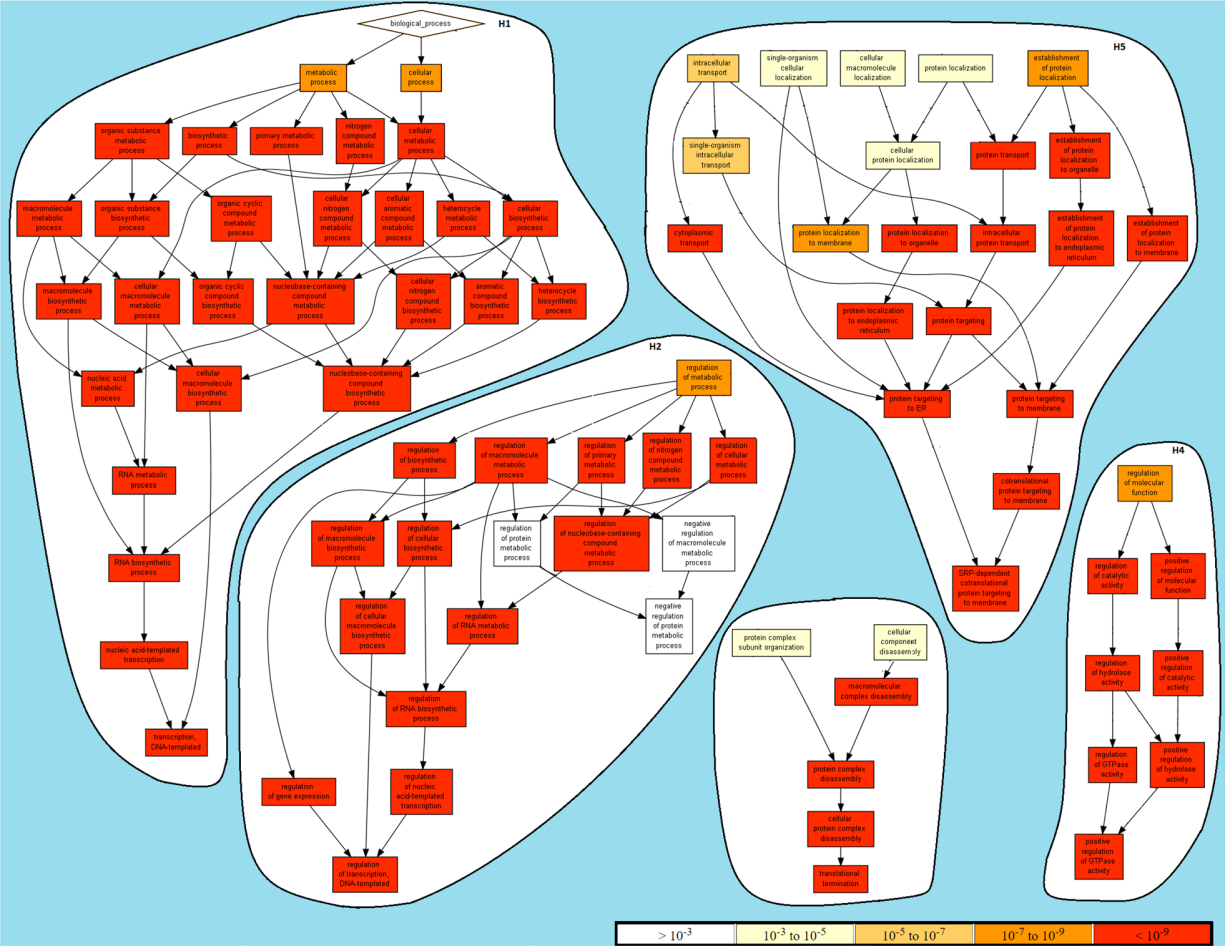
Taxonomy of enriched terms of BicNET’s modules discovered from human PPIs (see Table 4)

A subset of the overall modules collected from the application of BicNET over the selected biological networks is provided in Table 4. This table gathers modules with varying: tolerance to noise (overlapping threshold for merging procedures varied between 60 and 90 %), coherency assumption (dense, constant and order-preserving models) and coherency strength ($D_1$–$D_4$ with $\mathcal {L}$ = {−2,−1,1,2}, $Y_1$–$Y_5$ and $H_1$–$H_3$ with $\mathcal {L}$ = {1,2,3}, $Y_6$ and $H_4$ with $\mathcal {L}$ = {1,2,3,4}). All of the modules were discovered using multi-item assignments whenever values were found to be near a discretization boundary. The collected results show that all of BicNET’s modules had not only highly enriched terms, but also the enriched terms were found to be functionally related (taxonomically closed biological processes [54]). This observation suggests that the discovered modules are characterized by a cohesive set of putative biological functions. To support this observation, Figs. 18 and 19 provide an hierarchical visualization of some of the enriched terms (recurring to GOrilla tool [73]) for a subset of the discovered modules.

**Table 4:** Description of the biological role of an illustrative set of BicNET’s modules with varying properties

	ID	Homogeneity	$\sharp$Nodes $|I|\times |J|$	Putative functionality: group of enriched terms ($p<$1E−10)
STRING (yeast)	Y1	Dense (high noise-tolerance)	231 × 14	Metabolic processes with incidence on protein, peptide and amide metabolism and biosynthesis
	Y2	Dense (medium noise-tolerance)	217 × 9	Metabolism of nitrogen compounds and some organic substances
	Y3	Constant (few high $a_{ij}$)	103 × 8	Amino acid activation and tRNA metabolism for tRNA aminoacylation
	Y4	Constant (few high $a_{ij}$)	206 × 6	Organic acid metabolic process and its subterms
	Y5	Constant (few high or low $a_{ij}$)	55 × 7	Signal transduction and its subterms
	Y6	Constant (few high or low $a_{ij}$)	43 × 6	Phosphorylation related terms (with incidence on protein phosphorylation)
	Y7	Order-preserving	176 × 12	Transport of organic acids (with incidence on aminoacid transmembrane transport)
	Y8	Order-preserving	235 × 9	Oxidation-reduction process and metabolism of aminoacids. Assembly of ribonucleoprotein
	Y9	Order-pres. (few high $a_{ij}$)	146 × 8	Transport of molecules (highest enrichment found for drug transmembrane)
STRING (human)	H1	Dense (high noise-tolerance)	811 × 28	Multiple metabolic processes with incidence on transcription activity
	H2	Dense (high noise-tolerance)	787 × 25	Regulation of metabolic processes (both positive and negative regulation)
	H3	Constant (few high $a_{ij}$)	693 × 14	Regulation of intracellular signal transduction (over 20 highly enriched terms)
	H4	Constant (few high $a_{ij}$)	645 × 10	Regulation of molecular functions (incidence on catalytic activity)
	H5	Order-preserving	720 × 24	Establishment of protein localization (protein targeting to ER and membrane)
	H6	Order-preserving	733 × 29	Protein phosphorylation and its subterms
DryGIN	D1	Dense (high noise-tolerance)	28 × 17	Organelle localization (establishment of spindle and nuclear localization)
	D2	Constant (with pos&neg $a_{ij}$)	22 × 10	Chromatin remodeling and nucleosome organization
	D3	Constant (with pos&neg $a_{ij}$)	21 × 7	Transport processes for the establishment of protein localization
	D4	Constant (with pos&neg $a_{ij}$)	19 × 9	Regulation of growth (incidence on filamentous growth)
	D5	Order-preserving	39 × 7	Organelle and nucleous organization
	D6	Order-preserving	54 × 6	Regulation of cellular metabolic processes (both positive and negative regulation)

Three major observations are retrieved from the conducted analyzes. First, the combination of the dense model with the provided procedures to foster robustness leads to higher enrichment factors as key genes/proteins with subtler yet functional relevance were not excluded from the modules. Nevertheless, this form of coherency is mainly associated with broader biological processes, such as general metabolic and regulatory processes (see $Y_1$, $Y_2$, $H_1$ and $H_2$ modules). Second, the constant model is indicated to guarantee a focus on less trivial modules associated with a compact set of more specific biological processes. Modules $Y_3$–$Y_6$, $H_3$–$H_4$ and $D_2$–$D_4$ are example of the relevance of considering non-dense interactions since these interactions are often related with latent or secondary (yet critical) cellular functions. Third, the order-preserving coherency is associated with modules as large as the ones provided under the noise-tolerant dense coherency, yet with the additional benefit of enabling the presence of weaker interactions as long as their coherency among the nodes is respected.

#### Non-trivial modules

The provided modules in Table 4 already show unique properties that surpass some of the inherent limitations of the existing methods for network module discovery. Even so, BicNET can be used to further disclose less trivial modules, such as modules characterized by the presence of constant patterns with multiple symbols, symmetries and plaid effects. For this purpose, we parameterized BicNET with simple constraints ("BicNET: incorporating available domain knowledge" section) to guarantee that such modules appear in the output. Table 5 shows an illustrative set of such modules with significantly enriched terms. All of the illustrated modules show coherent patterns of interaction between nodes and have an average amount of 5–10 % of missing interactions. This analysis reinforces that BicNET is well positioned to find modules with varying size, coherency and quality. Illustrating, the constant modules $G_6$ and $G_7$ have, respectively, 25 and 50 nodes and distinct quality, being $G_7$ more tolerant to noisy interactions. Understandably, the number of nodes per module is naturally affected by the size and sparsity of the target network. The discovered modules clearly show non-trivial yet meaningful correlations (as they include interactions with coherent yet non-differential scores), whose relevance is pinpointed by the number of highly enriched terms after correction.

**Table 5:** Exclusivity and relevance of BicNET solutions: properties of found modules

	ID	Type	$\sharp$Nodes $|I|\times |J|$	Items	$\sharp$Terms *p* $<$1E−15	Notes
DryGIN	G1	Constant	18 × 9	{−4,..,−1}	27	Module with coherent strong (−4) and soft (−1) negative interactions
	G2	Symmetric	4 × 9	{−3,..,3}	13	Varying levels of strong (mainly positive) interactions ({$\pm$3,$\pm$2})
	G3	Symmetric	5 × 6	{−2,−1,1,2}	12	Module with either all positive or negative interactions per “row”-node ({$\pm$1,$\pm$2})
	G4	Constant	7 × 5	{1,2}	12	Module with coherent strong (2) and soft (1) positive interactions
	G5	Symmetric	7 × 5	{−2,−1,1,2}	11	Module with either all positive or negative interactions per “row”-node ({$\pm$1,$\pm$2})
	G6	Order	14 × 11	{−3,..,3}	25	Preserved precedences and co-occurrences per “row”-node before postprocessing
	G7	Order	42 × 8	{−2,−1,1,2}	50	Noise-tolerant module with mostly preserved orderings per “row”-node
STRING	S1	Order	155 × 14	{1,2,3,4}	169	Preserved precedences and co-occurrences per “row”-node before postprocessing
	S2	Constant	80 × 18	{1,2,3}	98	Module with mostly of non-dense interactions ({1,2})
	S3	Constant	83 × 10	{1,2}	93	Module with non-dense positive interactions before postprocessing ({1})
	S4	Constant	50 × 20	{1,2,3}	70	Module with non-dense positive interactions ({1,2}) before postprocessing
	S5	Constant	45 × 31	{1,2,3}	76	Module with mostly dense interactions (scores in {2,3})
	S6	Constant	55 × 85	{1,2}	143	Module with mostly dense interactions ({2})

Table 6 lists some of the enriched terms for the modules in Table 5, showing their functional coherence and role to unravel putative biological processes. Interestingly, as illustrated in Table 7, some of the identified modules are part of an additive plaid model (with in-between condition [21]). Illustrating, modules $G_6$ and $S_4$ share, respectively, 21 and 42 % of their interactions with modules $G_7$ and $S_2$ under a plaid assumption. Some properties of the two illustrative sets of overlapping modules are provided in Table 7. Without this assumption, only smaller modules (excluding key nodes) could be obtained, resulting in a lower enrichment of their terms.

**Table 6:** Illustrative set of biologically significant BicNET’s modules: description of the highly enriched terms in the modules presented in Table 5 [74, 75]

	ID	Terms description ($\sharp$)	$\sharp$Terms *p* $<$1E−15	$\sharp$Nodes
DryGIN	G1	Histone modification; regulation of histone H3-K79 methylation, histone H2B ubiquitination, H2B conserved C-terminal lysine ubiquitination, H3-K4 methylation (4)	6	27
	G2	Regulation of gluconeogenesis; glutamate metabolic and catabolic processes (2);nicotinamide riboside metabolic process; nicotinamide nucleotide biosynthetic process	6	13
	G3	Positive and negative regulation of transcription from RNA polymerase II; Invasive growth response to glucose limitation and hyperosmotic salinity response by regulating RNA polymerase II (5)	5	12
	G4	Meiotic anaphase I; activation of anaphase-promoting complex activity involved in meiotic cell cycle	4	12
	G5	Negative reg. of phospholipid biosynthesis; lipid homeostasis; isopropylmalate and oxaloacetate transport	4	11
	G6	Cotranslational protein targeting to membrane; protein insertion into mitochondrial membrane; protein import into peroxisome membrane; reg. sporulation; actin filament bundle assembly involved in cytokinesis	5	25
	G7	Acetate fermentation, acetyl-CoA biosynthesis (from acetate), reg. transcription on exit from mitosis	7	50
STRING	S1	Response to hypoxia; oxidation-dependent protein catabolic process; anaerobic respiration; age-dependent response to reactive oxygen species; cellular response to oxidative stress	36	169
	S2	Positive and negative reg. of mitotic and nuclear cell cycle, DNA replication, budding cell apical bud growth	16	98
	S3	Transport of aerobic electron, acetyl-CoA, vacuolar transmembrane, amine, transport (5); ribose phosphate metabolic process; D-ribose metabolic and catabolic processes (2)	22	93
	S4	Heterochromatin maintenance involved in chromatin silencing; sister chromatid segregation	6	70
	S5	Cytoplasmic and mitochondrial translation (4); regulation of translational fidelity; ADP biosynthesis	6	76
	S6	rRNA processing; separation, cleavage and maturation of SSU-rRNA (5); ribosomal (large subunit) biogenesis	14	143

**Table 7:** Sets of modules with meaningful overlapping areas (satisfying the in-between plaid assumption [21])

ID	Modules with meaningful overlapping regions	Pattern	$\sharp$Nodes $|I|\times |J|$	% Overlapping interactions
G6	G7 from Table 6 (orders preserved in overlapping regions before cumulative effect)	Order	42 × 8	21
	G8: tRNA re-export from nucleus; nuclear mRNA surveillance of mRNP export	Constant	12 × 10	62
	G9: More general module (background) including cellular responses to pH	Constant	41 × 6	16
S4	S2 from Table 6 (satisfying the relaxed additive model proposed in [21])	Constant	80 × 18	42
	S7: Telomere maintenance; translocation; protein import into nucleous	Constant	104 × 20	37
	S8: Response to ionizing radiation; ribose phosphate metabolic process	Constant	59 × 31	45
	S9: Positive regulation of mitochondrial translation in response to stress	Constant	50 × 20	89

The analysis of the enriched transcription factors (TFs) for each putative biological process in Table 6 further supports the previous functional enrichment analyzes. For this end, we retrieved the TFs that are more *representative* (high coverage of the genes in the module) and *significant* (high functional enrichment: *p* value$<$1E−3). Illustrating, $G_1$ has diverse TFs regulating different families of histones, such as Jhd1p [74]; in $G_4$ we found regulators of meiosis, including Sin3p [74]; the TFs of $G_7$ activate genes required for cytokinesis (exit from mitosis); in $S_1$ we found TFs associated with responses to oxygen-related stress, such as the activation of beta-oxidation genes by Pip2p [74]; proteins regulating $S_2$ respond to DNA damaging, such as Plm2p and Abf1p [75]; membrane sensors, such as Ure2p, are active in the regulation of genes in $S_3$; $S_4$ has proteins promoting the organization and remodeling of chromatin, including Abf1p, Plm2p and Rsc1p [75]; regulators of ribosomal biogenesis, such as Sfp1p (100 % representativity), and of its subunits, such as Cse2p [74], are core TFs for $S_6$.

#### Concluding note

When analyzing networks derived from knowledge-based repositories and literature (such as the networks from STRING [16]), the flexibility of coherence and noise-robustness is critical to deal with uncertainty and with the regions of the network where scores may be affected due to the unbalanced focus of research studies. When analyzing networks derived from data experiments (such as the GIs from DRYGIN [19]), the discovery of modules with non-necessarily strong interactions (e.g. given by the constant model) is critical to model less-predominant (yet key) biological processes, such as the ones associated with early stages of stimulation or disease.

## Conclusions and future work

This work tackles the task of biclustering large-scale network data to discover modules with non-dense yet meaningful coherency and robustness to noise. In particular, we explore the relevance of mining non-trivial modules in homogeneous and heterogeneous networks with quantitative and qualitative interactions. We proposed BicNET algorithm to extend state-of-the-art contributions on pattern-based biclustering with efficient searches on networks, thus enabling the exhaustive discovery of constant, symmetric and plaid models in biological networks. Additional strategies were further incorporated to retrieve modules robust to noisy and missing interactions, thus addressing the limitations of the existing exhaustive searches on networks. Finally, we have shown that BicNET can be assisted in the presence of background knowledge and user expectations.

Empirical evidence confirms the superiority of BicNET against peer biclustering algorithms able to discover non-dense regions. Contrasting with their efficiency bottlenecks, BicNET enables the analysis of dense networks with up to 50,000 nodes. Results on biological networks reveal its critical relevance to discover non-trivial yet coherent and biologically significant modules.

Five major directions are identified for upcoming research: (1) to gather missing and noisy interactions within the discovered modules to predict unknown interactions and to test the confidence (or adjust the score) of the weighted interactions within available biological networks; (2) to enlarge the conducted biological analysis to further establish relationships between modules and functions to support the characterization of biological molecules with yet unclear roles; (3) to explore the plaid model to identify and characterize hubs based on the overlapping interactions between modules, as well as the interactions within each of the two sets of interacting nodes per bicluster to further assess the connectivity, coherence and significance of modules; (4) to study the relevance of alternative forms of coherency given by biclustering algorithms with distinct homogeneity/merit functions [15]; and (5) to extend BicNET for the integrative analysis of GI and PPI networks and expression data in order to validate results and combine these complementary views either at the input, mining or output levels.

## Availability

The BicNET software (graphical and programmatic interfaces) and datasets can be accessed at https://web.ist.utl.pt/rmch/bicnet/.

## Footnotes

1 Consider the specific case where pattern-based biclustering is given by frequent itemset mining. Let $\mathcal {L}$ be a finite set of items, and *P* an itemset $P\subseteq \mathcal {L}$. A discrete matrix *D* is a finite set of transactions in $\mathcal {L}$, $\{P_1,..,P_n\}$. Let the *coverage*$\Phi _{P}$ of an itemset *P* be the set of transactions in *D* in which *P* occurs, $\{P_i \in D\mid P\subseteq P_i\}$, and its *support*$sup_P$ be the coverage size, $\mid \Phi _{P}\mid$. Given *D* and a minimum support threshold $\theta$, the *frequent itemset mining* (FIM) problem consists of computing: $\{P \mid P \subseteq \mathcal {L}, sup_P \ge \theta \}$. Given *D*, let a matrix *A* be the concatenation of *D* elements with their column (or row) indexes. Let $\Psi _P$ of an itemset *P* in *A* be its indexes, and $\Upsilon _P$ be its original items in $\mathcal {L}$. A set of *biclusters*$\cup _k (I_k,J_k)$ can be derived from a set of frequent itemsets $\cup _k P_k$ by mapping $(I_k,J_k)$=$B_k$, where $B_k$=$(\Phi _{P_k},\Psi _{P_k})$, to compose constant biclusters with coherency across rows (or $(I_k,J_k)$=$(\Psi _{P_k},\Phi _{P_k})$ for column-coherency) with pattern $\Upsilon _P$.

2 In the context of biological networks, biclustering has been also used to either validate or extract molecular interactions from biclusters discovered in gene expression and proteomic data [30–33]. This a rather distinct task that the target in this paper and thus out of the scope.

3 Tests and estimations based on the calculus of approximated statistical ratios described in http://www.pitt.edu/super1/ResearchMethods/Ricci-distributions-en.pdf (accessed January 2016).

4 BicNET software can be accessed at https://web.ist.utl.pt/rmch/bicnet/.

5 To run the experiments, we used: fabia package [67] from R, BicAT [68], BicPAM [15] and expander [20] softwares.

6 Sparse prior equation with decreasing sparsity until able to retrieve a non-empty set of biclusters.
